# Steering-Angle Prediction and Controller Design Based on Improved YOLOv5 for Steering-by-Wire System

**DOI:** 10.3390/s24217035

**Published:** 2024-10-31

**Authors:** Cunliang Ye, Yunlong Wang, Yongfu Wang, Yan Liu

**Affiliations:** 1 School of Mechanical Engineering and Automation, Northeastern University, Shenyang 110819, China; yecunliang123@126.com (C.Y.); wangyl@mail.neu.edu.cn (Y.W.); 2 School of Mechanical Engineering, Ningxia Institute of Science and Technology, Shizuishan 753000, China; 3 School of Automobile and Traffic Engineering, Liaoning University of Technology, Jinzhou 121001, China; dannieliuy@outlook.com

**Keywords:** steer-by-wire (SbW) systems, steering-angle prediction, autonomous vehicles (AVs), convolutional neural network (CNN), barrier Lyapunov function

## Abstract

A crucial role is played by steering-angle prediction in the control of autonomous vehicles (AVs). It mainly includes the prediction and control of the steering angle. However, the prediction accuracy and calculation efficiency of traditional YOLOv5 are limited. For the control of the steering angle, angular velocity is difficult to measure, and the angle control effect is affected by external disturbances and unknown friction. This paper proposes a lightweight steering angle prediction network model called YOLOv5Ms, based on YOLOv5, aiming to achieve accurate prediction while enhancing computational efficiency. Additionally, an adaptive output feedback control scheme with output constraints based on neural networks is proposed to regulate the predicted steering angle using the YOLOv5Ms algorithm effectively. Firstly, given that most lane-line data sets consist of simulated images and lack diversity, a novel lane data set derived from real roads is manually created to train the proposed network model. To improve real-time accuracy in steering-angle prediction and enhance effectiveness in steering control, we update the bounding box regression loss function with the generalized intersection over union (GIoU) to Shape-IoU_Loss as a better-converging regression loss function for bounding-box improvement. The YOLOv5Ms model achieves a 30.34% reduction in weight storage space while simultaneously improving accuracy by 7.38% compared to the YOLOv5s model. Furthermore, an adaptive output feedback control scheme with output constraints based on neural networks is introduced to regulate the predicted steering angle via YOLOv5Ms effectively. Moreover, utilizing the backstepping control method and introducing the Lyapunov barrier function enables us to design an adaptive neural network output feedback controller with output constraints. Finally, a strict stability analysis based on Lyapunov stability theory ensures the boundedness of all signals within the closed-loop system. Numerical simulations and experiments have shown that the proposed method provides a 39.16% better root mean squared error (RMSE) score than traditional backstepping control, and it achieves good estimation performance for angles, angular velocity, and unknown disturbances.

## 1. Introduction

### 1.1. Background

According to the latest World Health Organization (WHO) reports, approximately 1.3 million individuals lose their lives annually due to road accidents, with human errors accounting for an estimated 90% of all car crashes [1]. The statistics above have prompted the proposition of autonomous vehicles (AVs) as a means to mitigate human errors. AVs exemplify a burgeoning application of automotive technology, attracting significant attention from both academia and industry due to their advanced functionalities, such as scene recognition, path planning, and motion control, which offer substantial driving convenience. AVs are now emerging as an innocuous alternative to human drivers, thereby significantly reducing the annual loss of thousands of lives [2]. Noteworthy ongoing research efforts in managing the diverse challenges faced by AVs include advancements in human recognition, traffic analysis, road and lane detection, steering controls, and path planning. Simultaneously, the prediction and control of the steering angle play a pivotal role in the realm of autonomous vehicles, garnering significant attention from researchers, manufacturers, and insurance companies alike [3].

### 1.2. Related Research

In the last few years, significant advancements have been made in the field of AVs. The current research methodologies on AVs can be classified into two primary approaches: mediated perception and end-to-end driving. The mediated perception approach focuses on breaking the driving task down (e.g., perception, localization, planning, and control to ensure safe driving) into standardized modules. Subsequently, it employs rule-based methods to establish connections between these distinct modules. The mediated perception method, however, exhibits inherent limitations. The primary disadvantage lies in the intricate task of developing and maintaining interconnections between all modules within the system. The modularity paradigm may be compromised in different scenarios that require varying connections between modules [4]. Additionally, constructing and sustaining such a pipeline incurs significant costs despite extensive efforts over many years; thus, it is far from achieving complete autonomy [5]. Consequently, these approaches necessitate a substantial endeavor in designing architectures that integrate all system components and are frequently susceptible to error propagation throughout the entire pipeline [6,7].

Deep learning (DL) techniques are gaining increasing popularity and have emerged as a valuable tool across various industries, including the automotive sector, owing to their exceptional capability to extract image features. DL has significantly impacted AVs’ control, particularly in terms of steering-angle prediction, due to its efficient processing of unlabeled raw data and robust extraction of image features. These techniques have facilitated the emergence of the end-to-end driving approach, also called the behavior reflex approach, simplifying traditional subsystems considerably and alleviating the burden associated with vehicle modeling and control [8]. DL-based steering-angle prediction in the end-to-end driving approach offers several advantages, including error tolerance, rapid error identification, and an enhanced capability to manage unpredictable situations [9]. The work of [10] presents an intelligent driving assist system for the real-time prediction of the steering angle using DL and a raw data set collected from a real environment.

To date, many studies have employed deep learning techniques to predict steering angles and control lateral motion in autonomous vehicles. The earliest attempt at end-to-end driving can be traced back to ALVINN, where a three-layer, fully connected network was trained to predict the steering wheel angle based on camera and radar images [11]. Consequently, the researchers subsequently developed an end-to-end driving system specifically designed for off-road conditions. They extensively trained a six-layer convolutional network using a substantial amount of data to accurately simulate the obstacle avoidance behavior exhibited by human drivers [12], the small vehicle, known as DAVE. Ref. [13], expanded upon the DAVE system, training a three-camera model to perform steering control for a vehicle in a range of real-world driving scenarios. This work arguably brought end-to-end systems to the forefront of AV research. A convolutional neural network (CNN)-based end-to-end controller was proposed for steering autonomous vehicles [14]. The network was trained using road screenshots generated via the car simulator CARSIM and human driver steering angles. However, it is worth noting that the network architecture consists of only seven layers, which may limit its capacity, and the training data lack real-world derivation, leading to potential limitations in their generalization ability. The proposed framework in [15] introduces a multi-task demonstration learning (MT-LfD) approach, incorporating an end-to-end trainable network for emulating the driving commands of an expert demonstrator. Supervised auxiliary task prediction is employed to guide the primary task of predicting driving commands. A neural motion planner is proposed in [16] for autonomous driving in complex urban scenarios, encompassing traffic light processing, concessions, and interaction with multiple road users. To accomplish this, the authors developed a comprehensive model that utilizes raw liDAR data and high-definition maps as inputs to generate interpretable intermediate representations in the form of 3D detections and their future trajectories.

The ease of amassing extensive human driving data renders the end-to-end approach highly effective for straightforward tasks. However, intricate and infrequent traffic scenarios continue to pose challenges for this methodology [17]. From the relevant research above, the primary challenge in DL-based steering-angle prediction for AVs lies in the scarcity of real-world data sets, necessitating a heavy reliance on data generated from simulated environments. The limited depth of existing CNN-based networks has also constrained their training performance. Increasing the network depth can enhance performance. However, it also introduces challenges such as vanishing or exploding gradients and accuracy saturation, followed by rapid degradation [18].

Despite the numerous shortcomings and challenges that persist, end-to-end driving remains the most promising approach for AVs based on previous research findings. This approach offers significant advantages in terms of reducing hardware costs and research complexity compared to alternative methods. Furthermore, its incorporation of diverse data sets enables versatility across various scenarios [6]. The work of [19] presents an innovative approach to end-to-end steering-angle prediction and its control in electric power-steering (EPS) systems. The methodology integrates transfer learning-based computer vision techniques for prediction and control with fuzzy signature-enhanced fuzzy systems. Successfully applied transfer learning-based computer vision technology to extract corresponding visual data without the need for large data sets reduces data collection and the computer load. The experiment shows that the proposed model achieves good performance. Recently, with the rapid advancement of deep learning techniques, computer vision-based object detection, combined with deep learning, has gradually emerged as the predominant method in this field of AVs. This methodology eliminates the need for manual feature extraction and can be categorized into two main groups: two-stage object detection methods represented by Region with CNN (RCNN), Fast RCNN, Faster RCNN, and Mask RCNN [20], and one-stage object detection methods, represented by Single Shot MultiBox Detector (SSD) detection methods series, You Only Look Once (YOLO) detection methods series, and RetinaNet [21,22,23]. Among these approaches, YOLOv5 stands out as a cutting-edge representation due to its superior speed, enhanced detection accuracy, and reduced file size. Consequently, it finds extensive applications requiring precise object detection tasks in various domains. In this study, drawing inspiration from object detection methodologies, we transform the task of predicting steering angles into an object-detection problem and propose a novel steering controller.

A lot of research has been conducted on steering control in AVs. In order to obtain accurate tracking performance for SbW systems, for example, model-based linear quadratic feedback control [24], trajectory tracking control [25], and model predictive control [26] are widely used. Ref. [27] proposed an adaptive sliding-mode control method to improve control accuracy using self-aligning torque and friction as an external disturbance. Ref. [27] proposed a robust sliding-mode learning control scheme and designed a sliding-mode learning controller to drive the sliding-mode variable in order to converge the tracking error to zero. They ignore system-parameter uncertainty and external disturbances. Ref. [28] proposed a robust, adaptive, integral terminal sliding-mode control strategy based on extreme learning machines, which ensures the finite time convergence of errors and effectively estimates the total uncertainty in a system using a single hidden layer feed-forward network. The work of [29] presents a new, vertical noncontact angle sensor based on the electromagnetic induction principle and conducted nonlinear optimization. A sensor prototype was made and tested in a laboratory. The experimental results show that the nonlinearity of the sensor was significantly improved, making angle measurement more accurate. This provides accurate angle values in angle-control experiments, which helps improve control accuracy. However, although the above methods have achieved many results, there are still limitations in many areas. For example, it is necessary to accurately know the angular velocity signal, and in practice, additional sensors need to be installed, while using differential methods can amplify measurement noise. It cannot be guaranteed that the tracking error will always be within a specific range, and there may be sudden changes in the tracking error.

### 1.3. Motivation and Contributions

The remarkable advancements in automotive technology in recent years have been driven by a convergence of several interconnected trends, including the resurgence of deep learning, the rapid evolution of sensing devices and in-vehicle computing systems, the accumulation of annotated data, and significant breakthroughs in related research fields (particularly computer vision) [30]. The rapid progress of deep learning can be attributed to the emergence and extensive application of convolutional neural networks (CNNs) in computer vision and object detection, which has paved the way for autonomous vehicle development. However, numerous challenges still hinder the widespread adoption of AVs in practical applications. For instance, one such challenge pertains to vision-based steering-angle prediction for AV control and designing an effective lateral controller for dynamic path tracking in order to solve the existing problems in the corner prediction and tracking control of autonomous vehicles. For example, the traditional YOLOv5 applied to angle prediction entails high requirements for storage capacity and hardware. Unknown parameters are difficult to measure in steering-angle tracking control, the influence of external disturbance, and model uncertainty on the control effect.

In this paper, we address the challenges above by transforming the task of predicting steering angles into an object-detection problem, and we propose a steering controller. It imitates the driver’s perception of road conditions, decision-making, and steering implementation during vehicle operation. It utilizes a camera positioned behind the windshield to translate the observed lane image into a steering control signal. The selection of a DL-based object detection algorithm plays a crucial role in addressing the challenge of steering-angle prediction. Undoubtedly, YOLOv5 has garnered significant attention and demonstrated remarkable accomplishments. However, the constraints on storage capacity and hardware limitations present substantial challenges for deploying the full-scale YOLOv5 network model in vehicular equipment [31]. Therefore, due to its performance merits, YOLOv5s is selected as the network model for predicting steering angles. To achieve accurate steering-angle prediction while enhancing computational efficiency, a lightweight steering-angle prediction network model called YOLOv5Ms is proposed, based on YOLOv5s.

To mimic the driver’s behavior during autonomous driving, we categorized lane-line curves into 15 classes, based on their curvature, and we created a corresponding data set for training You Only Look Once version 5 with MobileNet version 3 (YOLOv5Ms) as a network model. During AVs’ operation, a camera mounted on the vehicle captures information about lane lines on the road. The trained YOLOv5Ms model is utilized to predict steering angles using these camera images. The processed steering angle is then transmitted to the steering controller via a serial port. Subsequently, our proposed controller receives this data through the serial port to enable appropriate steering control.

In order to ensure that the steering system of AVs can accurately and in a timely manner track the reference angle signal, the steering system’s modeling and the controller’s design are crucial. In practical engineering, there are many unknown parameters in the SbW system, such as friction torque, self-aligning torque, external disturbance, and angular velocity, that are difficult to measure. With the development of adaptive control technology, neural networks can approximate nonlinear functions with high accuracy. Meanwhile, the state observer and disturbance observer exert a good estimation effect for the variables that are difficult to measure and the external disturbance. Therefore, neural networks, state observers, and perturbation observers can be used to model SbW systems in order to improve model accuracy for better control accuracy. However, during the angle tracking of AVs, the tracking error should be guaranteed to be within a specific range, which may lead to accidents if sudden changes occur instantaneously. Therefore, we are motivated to explore a control method in an SbW system with model uncertainty, an external disturbance, and difficult-to-measure variables to improve the tracking accuracy while ensuring that the tracking error is always within a specific range.

The contributions of this paper can be summarized as follows:

1. The present paper proposes a lightweight steering-angle prediction network model, namely YOLOv5Ms, based on YOLOv5s to achieve model compression while maintaining detection accuracy and speed. To address the issues of low localization accuracy and slow regression speed in object detection boxes during training, we employ Shape-IoU_Loss as the regression loss function for bounding box improvement.
2. To ensure that the steering system of the autonomous vehicle responds quickly and accurately to the predicted steering-angle signal, an adaptive output feedback control scheme with output constraints based on a neural network is proposed in this paper. The advantage of this control method is that it can constrain the tracking error within a given range, improving the tracking accuracy. Meanwhile, the effect of model uncertainty, external disturbance, and unmeasurable variables on the system are compensated for.
3. To enhance the generalization capability of the proposed detection model in this study, we conducted an extended data collection experiment at Western Xia Park in Yinchuan City, building upon our previously created lane-line data set. To ensure consistency with the previously collected steering-angle information, we once again experimented using a Borgward SUV (BX5) vehicle. This time, we recorded 40 h of real-world driving videos and increased the number of images from 8000 to 20,000 in order to augment the data set’s diversity while maintaining an image size of 640×640×3 pixels. To our knowledge, this manually labeled data set utilizing ImageLabel is currently the most prominent one encompassing lane lines.

The rest of the paper is organized as follows. Section 2 presents the lane-line detection algorithm based on YOLOv5, while Section 3 details the data set’s organization and network training. The steering controller design and stability proof are discussed in Section 4, followed by an experimental evaluation in Section 5. Finally, conclusions are drawn in Section 6.

## 2. Methods

### 2.1. Lane-Line Detection Algorithm Description

The YOLOv5 algorithm, as the current representative deep learning algorithm in the YOLO series (Appendix A), exhibits exceptional performance in object detection with an accelerated training speed, enhanced accuracy, and broader applicability. Consequently, it holds significant potential for practical applications. Four models are derived based on network depth and feature map width, YOLOv5s, m, l, and x. Specifically, the four models share a consistent network structure comprising input, backbone, neck, and prediction-head components. The YOLOv5 network is categorized as a single-stage, end-to-end detection framework that treats object detection as a regression problem by predicting bounding boxes and class probabilities across the entire image. This model encompasses three crucial processes: boundary-box prediction, class prediction, and feature extraction. The well-designed network architecture enables flexibility and selectivity in diverse scenarios. Therefore, we adopt YOLOv5’s one-staged detection framework as the foundational architecture for steering-angle prediction.

The YOLOv5 network model has achieved quite good results, but one of the problems with the steering-angle prediction model-based YOLOv5 is that the model is huge, with many parameters and large calculation amounts, and it is difficult to apply in embedded devices; moreover, steering-angle prediction scenarios require low latency or response speed. Imagine what terrible things could happen if the detection model for steering-angle prediction were slow. So, working on a small but efficient detection model is crucial in these scenarios, at least for now, although the hardware will also become faster in the future. Therefore, in the steering-angle prediction model based on YOLOv5, there is still room for improvement and enhancement. Optimizing the model architecture by employing smaller convolutional cores and reducing the number of pooling layers can effectively mitigate the parameters and computational load. Different improvement methods can be adopted, based on specific application scenarios with varying detection difficulties. Hence, this paper proposes a lightweight steering-angle prediction network model based on YOLOv5s to achieve a streamlined design while minimizing FLOPs, parameter counts, and the overall model size without compromising detection accuracy.

The MobileNet network exhibits superior advantages in lightweight neural networks due to its reduced size, decreased computational requirements, and enhanced accuracy. Furthermore, mobile models have been constructed using progressively more efficient building blocks. MobileNetV3 is an amalgamation of three models: MobileNetV1’s depthwise separable convolutions, MobileNetV2’s inverted residual with a linear bottleneck, and MnasNet’s lightweight attention model based on squeeze and excitation (SE) structures [32,33,34]. To optimize its performance on mobile phone CPUs, MobileNetV3 underwent a hardware-aware network architecture search (NAS) complemented by the NetAdapt algorithm, followed by further enhancements through novel architectural advancements. Two versions of MobileNetV3 are available: Large, for high-performance platforms, and Small, for low-performance platforms. Instead of using partial 3×3 deep convolutions, MobileNetV3 introduces a deep convolution of size 5×5. The SE module and h-swish (HS) activation function are incorporated to enhance model accuracy. These techniques can be effectively combined to discover optimized models tailored to specific hardware platforms.

YOLOv5 is a widely adopted object-detection algorithm, while MobileNetV3 represents a lightweight architecture for convolutional neural networks. Leveraging the strengths of these two models, we propose YOLOv5Ms, a lightweight steering prediction network model based on YOLOv5s. This model efficiently and accurately performs target-detection tasks, achieving a balance between accuracy and latency, making it suitable for deployment on mobile devices.

**Figure 1:**
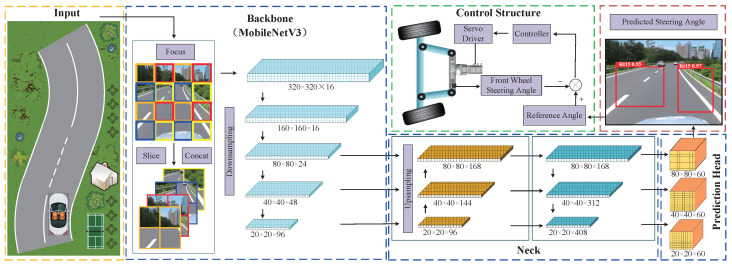
The lightweight steering-angle prediction network model, namely YOLOv5Ms, based on YOLOv5s and a control schematic network.

The schematic of steering-angle prediction and control based on the YOLOv5Ms network is illustrated in Figure 1. During autonomous driving, the vehicle-mounted camera captures road information in the form of images, which are then processed via a pre-trained YOLOv5 network. Subsequently, the predicted steering is transmitted to the steering controller. By mapping the steering-angle signal according to a predefined mapping relation, the input signal for the steering controller is obtained, resulting in corresponding steering outcomes through the actuation of the steering motor.

### 2.2. Improvement of YOLOv5Ms Network Architecture

In this section, we present a lightweight steering-angle prediction network model based on YOLOv5s. To enhance the performance of YOLOv5s, we have made two key improvements: replacing the original network backbone with MobileNetV3 and substituting the CIoU border regression function with Shape-IoU for better results. To reduce dimensionality without sacrificing features and computational efficiency, we have retained the focus structure at the input end of the network, as designed in YOLOv5. The resulting lightweight steering-angle prediction network model, named YOLOv5Ms, is built upon YOLOv5s, and its control schematic network is illustrated in Figure 1.

**Figure 2:**
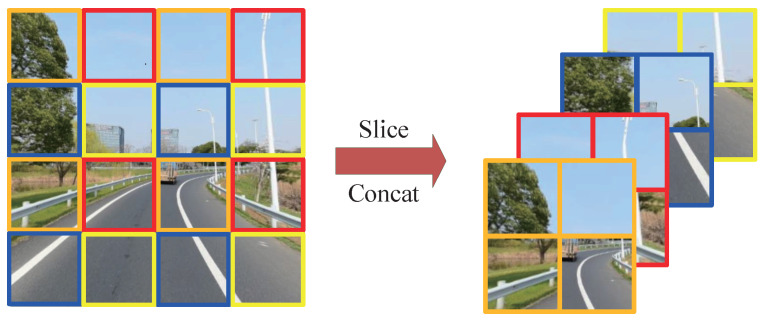
The specific slicing and concat principles.

Focus: YOLOv5 incorporates a focused structure at the network input to reduce dimensionality without compromising features and computational efficiency. The focus structure divides the preprocessed image into four parts using slicing operations, and it concatenates them. A 20% overlap area is introduced between the two image parts to ensure that lane lines are not disrupted. The specific principles of slicing and concatenation are illustrated in Figure 2. This focus module achieves downsampling while increasing channel dimensions, reducing FLOPs, and improving speed.

Backbone: To achieve a lightweight network architecture, we modified the backbone of yolov5s by incorporating the large and small modules of MobileNetV3 as alternative backbones. We named them YOLOv5Ms and YOLOv5Ml, respectively. The specifications for YOLOv5Ms and YOLOv5Ml of the backbone can be seen in Table 1 and Table 2. In the table, the input represents the shape transformation of each feature layer, while the operator signifies the block structure to be executed at each feature layer. The variables ‘size’ and ‘♯out’ respectively denote the number of channels after the inverse residual structure is applied to the bottleneck and the number of channels in the feature layer when it is input into the bottleneck (‘bk’). The term ‘SE’ indicates whether or not an attention mechanism, SE, is introduced in this module. In column six, ‘NL’ represents the type of activation function, where ‘HS’ denotes the h-swish, ‘RE’ represents ReLU, and ‘s’ indicates the stride.

**Figure 3:**
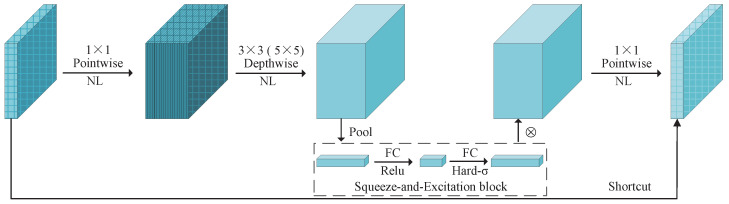
Bottleneck block structure.

The bottleneck structure is depicted in Figure 3, encompassing an inverse residual architecture that incorporates a linear bottleneck, depthwise separable convolution, and an SE attention mechanism. In the case of a stride value of 1 and input channels equal to output channels for the module, a shortcut connection is established between the input and output. Otherwise, subsequent operations follow the depthwise separable convolution operation, the SE attention mechanism, and the 1×1 point convolution operation. The depth separable convolution and squeeze-and-excitation block attention mechanism, SE, of the linear bottleneck are further described below.

(1) Depthwise separable convolution

The standard convolution operation takes an input tensor, *F*, of size $D_{F}\times D_{F}\times M$ and applies a convolutional kernel, $K\in R^{k\times k\times M\times N}$, to generate an output tensor, *G*, of size $D_{G}\times D_{G}\times N$. Here, $D_{F}$ represents the spatial width and height of a square input feature map, *M* denotes the number of input channels (input depth), and $D_{G}$ corresponds to the spatial width and height of a square output feature map. Finally, *N* signifies the number of output channels (output depth) [35].

The standard convolutional layer is parameterized by a square convolution kernel, *K*, with spatial dimensions of $D_{K}\times D_{K}\times M\times N$, where *M* represents the number of input channels, and *N* represents the number of output channels, as defined previously. Assuming stride one and padding, the computation for the output feature map in standard convolution can be expressed as follows:

(1) $$\begin{array}{c} \begin{array}{cc} \; & G_{k,l,n}=\sum_{i,j,m}K_{i,j,m,n}\cdot F_{k+i-1,l+j-1,m} \end{array} \end{array}$$

Standard convolutions have the computational cost of

(2) $$\begin{array}{c} \begin{array}{cc} \; & C_{s-cost}=D_{F}\times D_{F}\times D_{K}\times D_{K}\times M\times N \end{array} \end{array}$$

It is evident that the computational cost is multiplicatively dependent on various factors, including the number of input channels (*M*) and output channels (*N*), the kernel size ($D_{K}\times D_{K}$), and the feature map size ($D_{F}\times D_{F}$).

**Figure 4:**
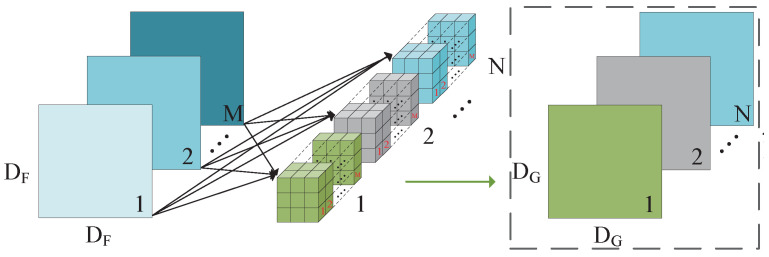
Standard convolution architecture.

**Figure 5:**
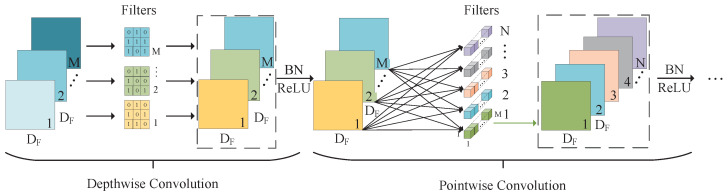
Depthwise separable convolution architecture.

Depthwise separable convolutions serve as a fundamental component in numerous efficient neural network architectures, and we employed them in our current study as well. This form of factorized convolutions breaks a standard convolution down into two parts: depthwise convolution, which applies a single filter per input channel for lightweight filtering, and pointwise convolution (a 1 × 1 convolution), responsible for generating new features by computing linear combinations of the input channels [32]. This factorization significantly reduces the computation and model size. The standard and depthwise separable convolution principles are illustrated in Figure 4 and Figure 5.

Depthwise convolutions are employed to apply a singular filter for each input channel, while pointwise convolution, represented as a simple 1×1 convolution, is subsequently utilized to generate a linear combination of the output from the depthwise layer. The  bottleneck block incorporates both batch normalization and rectified linear unit (ReLU) nonlinearities in both layers.

The depthwise convolution operation establishes a one-to-one correspondence between the convolution kernel and channel. Each channel is convolved with only one specific convolution kernel, generating feature map channels that exactly match the number of input channels. Mathematically, depthwise convolution can be represented as employing a single filter per input channel (input depth):

(3) $$\begin{array}{c} \begin{array}{cc} \; & {\tilde{G}}_{k,l,m}=\sum_{i,j}{\tilde{K}}_{i,j,m}\cdot F_{k+i-1,l+j-1,m} \end{array} \end{array}$$

where $\tilde{K}$ is the depthwise convolutional kernel of size $D_{K}\times D_{K}\times M$, and the $m_{th}$ filter in $\tilde{K}$ is applied to the $m_{th}$ channel in *F* in order to produce the $m_{th}$ channel of the filtered output feature map $\tilde{G}$. Hence, depthwise convolution has a computational cost of

(4) $$\begin{array}{c} \begin{array}{cc} \; & C_{Dw-cost}=D_{F}\times D_{F}\times D_{K}\times D_{K}\times M \end{array} \end{array}$$

The pointwise convolution operations resemble standard convolution operations, as they employ a 1×1×M convolution kernel, where *M* represents the number of channels in the preceding layer. Consequently, this convolution operation amalgamates the previous step’s map in a depth-wise manner with appropriate weights to generate a novel feature map. Multiple output feature maps are produced for each convolution kernel. Pointwise convolution and depthwise separable convolutions have the computational cost of

(5) $$\begin{array}{ll} C_{Pw-cost} & =D_{F}\times D_{F}\times 1\times 1\times M\times N\\ & =D_{F}\times D_{F}\times M\times N \end{array}$$

(6) $$\begin{array}{c} \begin{array}{cc} \; & C_{dp-cost}={D_{F}}^{2}\times {D_{K}}^{2}\times M+{D_{F}}^{2}\times M\times N \end{array} \end{array}$$

By expressing convolution as a two-step process of filtering and combining, we achieve a reduction in the computation of

(7) $$\begin{array}{c} \begin{array}{cc} \; & \frac{{D_{F}}^{2}\times {D_{K}}^{2}\times M+{D_{F}}^{2}\times M\times N}{{D_{F}}^{2}\times {D_{K}}^{2}\times M\times N}=\frac{1}{N}+\frac{1}{{D_{K}}^{2}} \end{array} \end{array}$$

**Figure 6:**
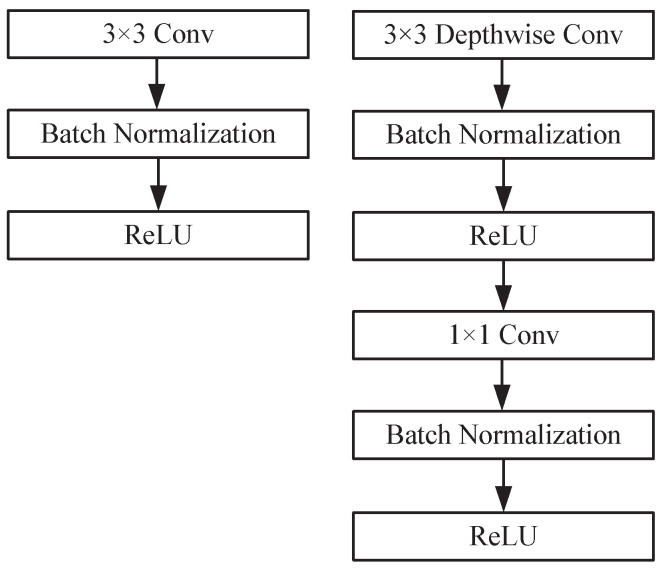
(**Left**): standard convolutional layer with batchnorm and ReLU. (**Right**): depthwise, separable convolution with depthwise and pointwise layers, followed by batchnorm and ReLU.

The depthwise separable convolution effectively reduces the computation compared to traditional layers by a factor of approximately $k^{2}$. When a 3×3 convolution kernel is assumed to be utilized in the depthwise separable convolution, it requires only 8 to 9 times less computation than the standard convolution without considering bias while maintaining a negligible decrease in accuracy. Figure 6 illustrates the comparison between standard convolution and depthwise, separable convolution with the batch norm and ReLU.

(2) Squeeze-and-excitation blocks

CNNs have emerged as valuable models for processing diverse visual tasks, making them the fundamental network structure employed in our study [36]. Recent research has demonstrated that incorporating attention mechanisms into CNN can enhance feature representations by effectively capturing spatial correlations. The attention mechanism is inspired by humans’ ability to process external information, where individuals selectively attend to relevant information while filtering out irrelevant stimuli due to the limited processing capacity of the human brain [37,38].

In order to obtain a more robust representation, only the most significant attributes for predicting the steering angle in images captured via the front-mounted camera during driving are retained, thereby enhancing performance. We introduce a novel architectural unit called the squeeze-and-excitation (SE) block that explicitly models interdependencies between convolutional feature channels to improve network representations [39].

**Figure 7:**
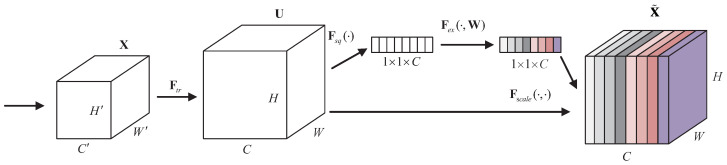
The structure of the SE building block.

The structure of the SE building block is depicted in Figure 7. Assume that input feature maps of the SE block X have shape $W^{\prime }\times H^{\prime }\times C^{\prime }$, where $W^{\prime }$ is the width, $H^{\prime }$ is height, and $C^{\prime }$ is the channels of feature maps. $F_{tr}$ can be thought of as a standard convolution operator. $F_{tr}$ maps an input $X\in R^{W^{\prime }\times H^{\prime }\times C^{\prime }}$ to feature maps $U\in R^{W\times H\times C}$. In the notation that follows, we use $K=[k_{1},k_{2},\ldots ,k_{C}]$ to denote the learned set of filter kernels, where $k_{c}$ refers to the parameters of the *c*th filter. We can then write the outputs as $U=[u_{1},u_{2},\ldots ,u_{C}]$, where $u_{c}$ can be expressed mathematically as follows:

(8) $$\begin{array}{c} u_{c}=k_{c}*X=\sum_{m=1}^{C^{\prime }}k_{c}^{m}*x^{m} \end{array}$$

Here, $k_{c}=\left[k_{c}^{1},k_{c}^{2},\cdots ,k_{c}^{C^{\prime }}\right]$, ∗ denotes a convolution operation, $X=[x^{1},x^{2},\cdots ,x^{C^{\prime }}]$, and $u_{c}\in R^{H\times W}$. $k_{c}^{m}$ is a 2D spatial kernel representing a single channel of $k_{c}$ that acts on the corresponding channel of X. To enhance the clarity of notation, the inclusion of bias terms has been omitted.

In addition to high-level channel relationships, convolutional models inherently involve implicit and localized channel dependencies. We anticipate that explicitly modeling interdependencies between channels will enhance the learning of convolutional features, thereby improving the network’s sensitivity to informative features that subsequent transformations can effectively utilize. Consequently, we aim to provide it with access to global information and recalibrate filter responses in two steps, namely squeezing and excitation, before they are fed into the next transformation. The SE attention mechanism enables the feature map to access global information and recalibrate the filter response through squeezing and excitation steps before proceeding with subsequent transformations [39].

After $F_{tr}$ mapping an input, $X\in R^{W^{\prime }\times H^{\prime }\times C^{\prime }}$, to feature maps, $U\in R^{W\times H\times C}$, to solve the problem of utilizing channel dependencies, we squeeze global spatial information into a channel description by using global average pooling operations to generate channel-wise statistics. The function of this descriptor is to generate an embedment of the corresponding global distribution of channel features, allowing all layers to use information from the global receptive domain (receptive field) of the network. Formally, the statistic $z\in R^{C}$ is generated via a shrinking of U through its spatial dimension H×W, and the *c*-th element of z is calculated as follows:

(9) $$\begin{array}{c} z_{c}=F_{sq}\left(u_{c}\right)=\frac{1}{H\times W}\sum_{i=1}^{H}\sum_{j=1}^{M}u_{c}\left(i,j\right) \end{array}$$

Here, $F_{sq}$ represents the global average pooling of channels.

To take advantage of the information aggregated in the squeeze operation, the second operation (excitation operation) is performed to capture the channel-wise dependency fully. To limit the complexity of the model and facilitate generalization, parameterized gating mechanisms are parameterized by forming a bottleneck containing two fully connected layers (FCs) around the nonlinearity. The first fully connected layer compresses *C* channels into $\frac{C}{r}$ channels to reduce the computational load, and a ReLU nonlinear activation, δ, is then used. The second fully connected layer returns to the number of the channel dimension to the original *C* channels and then obtains the weight s through sigmoid activation, σ. Therefore, the weight, W, of *C* feature maps in U can be expressed as follows:

(10) $$\begin{array}{c} s=F_{ex}\left(z,W\right)=\sigma \left(g\left(z,W\right)\right)=\sigma \left(W_{2}\delta \left(W_{1}z\right)\right) \end{array}$$

where *r* refers to the proportion of compression, and we take r=4. $s\in R^{1\times 1\times C}$, $W_{1}\in R^{\frac{C}{r}\times C}$, $W_{2}\in R^{C\times \frac{C}{r}}$. The final output of the squeeze-and-excitation block is obtained by rescaling U using the activation s:

(11) $$\begin{array}{c} {\tilde{x}}_{c}=F_{scale}\left(u_{c},s_{c}\right)=s_{c}u_{c} \end{array}$$

where $\tilde{X}=\left[{\tilde{x}}_{1},{\tilde{x}}_{2},\ldots ,{\tilde{x}}_{C}\right]$, and $F_{scale}\left(u_{c},s_{c}\right)$ refer to channel-wise multiplication between the scalar $s_{c}$ and the feature map $u_{c}\in R^{H\times W}$. SE blocks intrinsically introduce dynamics conditioned on the input, which can be regarded as a self-attention function on channels whose relationships are not confined to the local receptive field that the convolutional filters are responsive to. The SE blocks are also a flexible plug-and-play module. When a bottleneck module in the network backbone does not use SE, the  bottleneck module can carry out the normal convolution process.

### 2.3. Loss Function

Object detection is a fundamental challenge in computer vision tasks, and bounding-box regression plays a pivotal role in accurately predicting the location of target objects. In our proposed YOLOv5Ms network, we make predictions for boxes at three different scales [40]. The final scale generates a 3D tensor that encodes information about bounding boxes, objectness scores, and class predictions. At each scale, we predict three boxes, resulting in a tensor of size D×D×[3×(4+1+15)] to account for the four bounding box offsets, one objectness prediction, and fifteen class predictions. Herein, *D* represents the grid size of the detection head.

**Figure 8:**
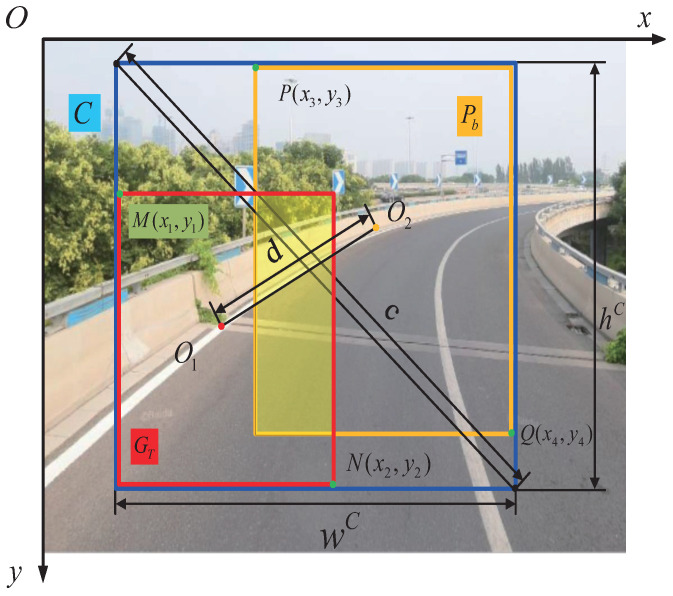
Schematic diagram of the bounding-box regression loss function.

Regarding the evaluation metric for bounding-box regression, conventional object detectors typically employ the mean square error (MSE) to directly regress the center-point coordinates, height, and width of the bounding box (BBox). However, treating each point of the BBox as independent variables when estimating their coordinate values fails to consider the holistic nature of the object itself. To address this issue and overcome the neglect of object integrity, a novel intersection over union (IoU) loss function was proposed for bounding boxes [41]. This loss function takes into consideration the coverage of both predicted and ground-truth bounding-box areas. It jointly regresses all bound variables as a unified unit by directly enforcing the maximal overlap between the predicted bounding box and the ground truth, as illustrated in Figure 8. The schematic diagram in Figure 8 demonstrates the process of bounding-box regression for this loss function. The X-axis represents the horizontal direction, while the Y-axis represents the vertical direction. The computation of the IoU loss involves calculating four coordinate points of the BBox by comparing them with the ground truth and then connecting these generated results to form a complete code. The most popular metric of IoU is as follows:

(12) $$\begin{array}{c} \begin{array}{cc} \; & IoU=\frac{|G_{T}⋂P_{b}|}{|G_{T}⋃P_{b}|} \end{array} \end{array}$$

(13) $$\begin{array}{c} \begin{array}{cc} \; & G_{T}=\left(x_{2}-x_{1}\right)\times \left(y_{2}-y_{1}\right) \end{array} \end{array}$$

(14) $$\begin{array}{c} \begin{array}{cc} \; & P_{b}=\left(x_{4}-x_{3}\right)\times \left(y_{4}-y_{3}\right) \end{array} \end{array}$$

where $G_{T}$ is the ground truth area with lane lines, as labeled by our own bounding box, and $P_{b}$ is the predicted area of the object bounding box. $M(x_{1},y_{1})$, $N(x_{2},y_{2})$, $P(x_{3},y_{3})$, and $Q(x_{4},y_{4})$ are the left top and bottom right corners of the two bounding boxes, respectively. The intersection of $G_{T}$ and $P_{b}$ is calculated via Equation (15):

(15) $$\begin{array}{c} \begin{array}{cc} \; & |G_{T}⋂P_{b}|=|(min\left(x_{2},x_{4}\right)-max\left(x_{1},x_{3}\right)\\ \; & \;\;\;\;\;\;\;\;\;\;\;\;\;\;\times (min\left(y_{2},y_{4}\right)-max\left(y_{1},y_{3}\right)| \end{array} \end{array}$$

Therefore, the IoU loss function can be expressed as follows:

(16) $$\begin{array}{c} \begin{array}{cc} \; & L_{IoU}=1-\frac{|G_{T}⋂P_{b}|}{|G_{T}⋃P_{b}|} \end{array} \end{array}$$

However, the IoU loss only works when the bounding boxes have overlap and would not provide any moving gradient for non-overlapping cases, and then the generalized IoU loss (GIoU) was proposed in [42], adding a penalty term C expressed as follows:

(17) $$\begin{array}{ll} |C|= & |[\max\left(x_{2},x_{4}\right)-\min\left(x_{1},x_{3}\right)]\times \\ & \;[\max\left(y_{2},y_{4}\right)-\min\left(y_{1},y_{3}\right)]| \end{array}$$

The GIoU loss function can be expressed as follows:

(18) $$\begin{array}{c} \begin{array}{cc} \; & L_{GIoU}=1-IoU+\frac{|C-G_{T}⋃P_{b}|}{\left|C\right|} \end{array} \end{array}$$

where *C* is the smallest box covering $G_{T}$ and $P_{b}$, as is shown in Figure 8. The GIoU loss includes the shape and orientation of the object, in addition to the coverage area. They proposed finding the smallest-area BBox that can simultaneously cover the predicted BBox and ground-truth BBox and using this BBox as the denominator to replace the denominator originally used in the IoU loss.

Introducing the penalty term facilitates the movement of the predicted box towards the target box in non-overlapping scenarios. Despite its ability to alleviate the gradient vanishing issue for such cases, GIoU still exhibits certain limitations. When bounding boxes enclose objects, the GIoU loss degrades to the IoU loss. A new approach called the distance-IoU (DIoU) loss is proposed for bounding-box regression to address this limitation. The DIoU loss incorporates a penalty term into the IoU loss to directly minimize the normalized distance between the central points of two bounding boxes, resulting in significantly faster convergence compared to the GIoU loss [43]. The objective of the DIoU loss is to simultaneously consider both the overlap area and the central-point distance when evaluating bounding boxes. Generally, the DIoU loss can be defined as follows:

(19) $$\begin{array}{c} \begin{array}{cc} \; & L_{DIoU}=1-IoU+\frac{\rho^{2}\left(O_{1},O_{2}\right)}{c^{2}} \end{array} \end{array}$$

As shown in Figure 8, $O_{1}$ and $O_{2}$ denote the central points of $G_{T}$ and $P_{b}$, respectively. $\rho \left(\cdot \right)=∥O_{2}-O_{1}{∥}_{2}$ is the Euclidean central distance of the predicted BBox and ground-truth BBox, and *c* is the diagonal length of *C*, which is the smallest enclosing box covering the two boxes.

The loss function for bounding-box regression should incorporate three crucial geometric measures, namely the overlap area, central-point distance, and aspect ratio. Consequently, building upon the DIoU loss, ref. [43] proposed a comprehensive IoU (CIoU) loss that integrates these aforementioned geometric measures. This novel approach facilitates faster convergence and yields superior performance compared to both IoU and GIoU losses. Then, the loss function can be defined as follows:

(20) $$\begin{array}{c} \begin{array}{cc} \; & L_{CIoU}=1-IoU+\frac{\rho^{2}\left(O_{1},O_{2}\right)}{c^{2}}+\alpha \upsilon \end{array} \end{array}$$

(21) $$\begin{array}{c} \begin{array}{cc} \; & \upsilon =\frac{4}{\pi^{2}}{\left(\arctan\frac{w^{G_{T}}}{h^{G_{T}}}-\arctan\frac{w^{P_{b}}}{h^{P_{b}}}\right)}^{2} \end{array} \end{array}$$

(22) $$\begin{array}{c} \begin{array}{cc} \; & \alpha =\frac{\upsilon }{(1-IoU)+\upsilon } \end{array} \end{array}$$

where α is a positive trade-off parameter, and υ measures the consistency of the aspect ratio. $w^{G_{T}}$ and $h^{G_{T}}$ are the width and height of the ground-truth bounding box, while $w^{P_{b}}$ and $h^{P_{b}}$ represent the width and height of the predicted bounding box. The gradient of υ with reference to $w^{P_{b}}$ and $h^{P_{b}}$ should be specified as follows:

(23) $$\begin{array}{c} \begin{array}{cc} \; & \frac{\partial \upsilon }{\partial w^{P_{b}}}=\frac{8}{\pi^{2}}\left(\arctan\frac{w^{G_{T}}}{h^{G_{T}}}-\arctan\frac{w^{P_{b}}}{h^{P_{b}}}\right)\\ \; & \;\;\;\;\;\;\;\;\;\times \frac{h^{P_{b}}}{{w^{P_{b}}}^{2}+{h^{P_{b}}}^{2}} \end{array} \end{array}$$

(24) $$\begin{array}{c} \begin{array}{cc} \; & \frac{\partial \upsilon }{\partial h^{P_{b}}}=-\frac{8}{\pi^{2}}\left(\arctan\frac{w^{G_{T}}}{h^{G_{T}}}-\arctan\frac{w^{P_{b}}}{h^{P_{b}}}\right)\\ \; & \;\;\;\;\;\;\;\;\;\;\times \frac{w^{P_{b}}}{{w^{P_{b}}}^{2}+{h^{P_{b}}}^{2}} \end{array} \end{array}$$

The dominator ${w^{P_{b}}}^{2}+{h^{P_{b}}}^{2}$ is usually a small value for the cases $w^{P_{b}}$ and $h^{P_{b}}$, with a range of [0, 1], which is likely to yield gradient explosion, and thus, in our implementation, the dominator ${w^{P_{b}}}^{2}+{h^{P_{b}}}^{2}$ is simply removed for stable convergence, through which the step size $\frac{1}{{w^{P_{b}}}^{2}+{h^{P_{b}}}^{2}}$ is replaced with 1, and the gradient direction is still consistent with Equations (23) and (24).

Since the υ only reflects the discrepancy of the aspect ratio, the CIoU loss may optimize the similarity unreasonably. To address the above problems, the work of [44] revised the CIoU loss and proposed a more efficient version of IoU (EIoU), the EIoU loss, which is defined as follows:

(25) $$\begin{array}{ll} L_{EIoU} & =1-IoU+\frac{\rho^{2}\left(O_{1},O_{2}\right)}{c^{2}}+\frac{\rho^{2}\left(w^{G_{T}},w^{P_{b}}\right)}{{\left(w^{C}\right)}^{2}}\\ & +\frac{\rho^{2}\left(h^{G_{T}},h^{P_{b}}\right)}{{\left(h^{C}\right)}^{2}} \end{array}$$

where $h^{C}$ and $w^{C}$ are the width and height of the smallest enclosing box, C, covering the two boxes.

Based on previous research, SIoU further considers the influence of the angle between the bounding boxes on the bounding box regression, which aims to accelerate the convergence process by decreasing the angle between the anchor box and the $G_{T}$ box, which is the horizontal or vertical direction [45]. Its definition is as follows:

(26) $$\begin{array}{c} \begin{array}{cc} \; & L_{SIoU}=1-IoU+\frac{\Delta +\Omega }{2} \end{array} \end{array}$$

(27) $$\begin{array}{c} \begin{array}{cc} \; & \Delta =\sum_{t=w^{P_{b}},h^{P_{b}}}\left(1-e^{-\gamma \rho_{t}}\right),\gamma =2-\Lambda \end{array} \end{array}$$

(28) $$\begin{array}{c} \begin{array}{cc} \; & \Lambda =\sin(2\sin^{-1}\frac{min(|x_{c}^{G_{T}}-x_{c}^{P_{b}}|,|y_{c}^{G_{T}}-y_{c}^{P_{b}}|)}{\sqrt{{\left(x_{c}^{G_{T}}-x_{c}^{P_{b}}\right)}^{2}+{\left(y_{c}^{G_{T}}-y_{c}^{P_{b}}\right)}^{2}}+\epsilon }) \end{array} \end{array}$$

(29) $$\begin{array}{c} \begin{array}{c} \left\{\begin{array}{c} \rho_{w^{P_{b}}}={\left(\frac{x_{c}^{G_{T}}-x_{c}^{P_{b}}}{w^{C}}\right)}^{2}\\ \rho_{h^{P_{b}}}={\left(\frac{y_{c}^{G_{T}}-y_{c}^{P_{b}}}{h^{C}}\right)}^{2} \end{array}\right. \end{array} \end{array}$$

(30) $$\begin{array}{c} \begin{array}{cc} \; & \Omega =\sum_{t=w^{P_{b}},h^{P_{b}}}{\left(1-e^{-\varpi_{t}}\right)}^{\theta },\theta =4 \end{array} \end{array}$$

where the value of θ defines how much the shape costs and its unique value for each data set. The value of θ is a significant term in this equation; it controls how much attention should be paid to the cost of the shape. If a value of θ is set to be 1, it will immediately optimize the shape, thus harming the free movement of a shape. To calculate the value of θ, the genetic algorithm is used for each data set; experimentally, the value of θ near to 4, and the range that the author defined for this parameter is from 2 to 6.

(31) $$\begin{array}{c} \begin{array}{cc} \; & \varpi_{w^{P_{b}}}=\frac{|w^{G_{T}}-w^{P_{b}}|}{max(w^{G_{T}},w^{P_{b}})}\\ \; & \varpi_{h^{P_{b}}}=\frac{|h^{G_{T}}-h^{P_{b}}|}{max(h^{G_{T}},h^{P_{b}})} \end{array} \end{array}$$

In conclusion, the previous bounding box regression methods mainly achieve more accurate regression by adding new geometric constraints on top of IoU. The above methods considered the influence of the distance, shape, and angle of the $G_{T}$ box and the anchor box on the bounding-box regression but neglected the fact that the shape and scale of the bounding box itself will also exert influences on the bounding-box regression. In order to further improve the accuracy of the regression, the authors proposed a new generation of the bounding regression loss: Shape-IoU in [46].

(32) $$\begin{array}{c} \begin{array}{cc} \; & w_{wt}=\frac{2\times {\left(w^{G_{T}}\right)}^{scale}}{{\left(w^{G_{T}}\right)}^{scale}+{\left(h^{G_{T}}\right)}^{scale}} \end{array} \end{array}$$

(33) $$\begin{array}{c} \begin{array}{cc} \; & h_{wt}=\frac{2\times {\left(h^{G_{T}}\right)}^{scale}}{{\left(w^{G_{T}}\right)}^{scale}+{\left(h^{G_{T}}\right)}^{scale}} \end{array} \end{array}$$

(34) $$\begin{array}{c} \begin{array}{cc} \; & D_{shape}=h_{wt}\times \frac{{\left(x_{c}^{P_{b}}-x_{c}^{G_{T}}\right)}^{2}}{c^{2}}+w_{wt}\times \frac{{\left(y_{c}^{P_{b}}-y_{c}^{G_{T}}\right)}^{2}}{c^{2}} \end{array} \end{array}$$

(35) $$\begin{array}{c} \begin{array}{cc} \; & \Omega_{shape}=\sum_{t=w^{P_{b}},h^{P_{b}}}{\left(1-e^{-\varpi_{t}}\right)}^{\theta },\theta =4 \end{array} \end{array}$$

(36) $$\begin{array}{c} \begin{array}{c} \left\{\begin{array}{c} \varpi_{w^{P_{b}}}=h_{wt}\times \frac{|w^{G_{T}}-w^{P_{b}}|}{max(w^{G_{T}},w^{P_{b}})}\\ \varpi_{h^{P_{b}}}=w_{wt}\times \frac{|h^{G_{T}}-h^{P_{b}}|}{max(h^{G_{T}},h^{P_{b}})} \end{array}\right. \end{array} \end{array}$$

where scale is the scale factor, which is related to the scale of the target in the data set, and $w_{wt}$ and $h_{wt}$ are the weight coefficients in the horizontal and vertical directions, respectively, whose values are related to the shape of the GT box. $D_{shape}$ is the distance shape. Its corresponding bounding box regression loss is as follows:

(37) $$\begin{array}{c} \begin{array}{cc} \; & L_{Shape-IoU}=1-IoU+D_{shape}+0.5\times \Omega_{shape} \end{array} \end{array}$$

YOLOv5 previously used GIoU; for the above reasons, in this paper, we use $L_{Shape-IoU}$ as the bounding-box regression-loss function. Moreover, the excellent performance of Shape-IoU as a bounding-box regression-loss function has been verified in [46].

The loss function used in the training of the YOLOv5Ms in the prediction of the steering angle mainly included the bounding-box location loss($L_{Shape-IoU}$), confidence loss ($L_{confidence}$), and classification loss ($L_{class}$), as follows:

(38) $$\begin{array}{c} \begin{array}{cc} \; & Loss=L_{Shape-IoU}+L_{confidence}+L_{class} \end{array} \end{array}$$

(39) $$\begin{array}{c} \begin{array}{cc} \; & Loss=1-IoU+D_{shape}+0.5\times \Omega_{shape}-\\ \; & \sum_{i=0}^{S^{2}}\sum_{j=0}^{B}I_{ij}^{obj}\left[\;\tilde{C_{i}^{j}}\log\left(\overset{´}{C_{i}^{j}}\right)+\left(1-\tilde{C_{i}^{j}}\right)\log\left(1-\overset{´}{C_{i}^{j}}\right)\right]-\\ \; & \lambda_{noobj}\sum_{i=0}^{S^{2}}\sum_{j=0}^{B}I_{ij}^{noobj}\left[\;\tilde{C_{i}^{j}}\log\left(\overset{´}{C_{i}^{j}}\right)+\left(1-\tilde{C_{i}^{j}}\right)\log\left(1-\overset{´}{C_{i}^{j}}\right)\right]\\ \; & -\sum_{i=0}^{S^{2}}I_{i}^{obj}\sum_{\tilde{c}\in class}[\;{\tilde{p}}_{i}\left(\tilde{c}\right)\log\left({\overset{´}{p}}_{i}\left(\tilde{c}\right)\right)+\\ \; & \;\left(1-{\tilde{p}}_{i}\left(\tilde{c}\right)\right)\log\left(1-{\overset{´}{p}}_{i}\left(\tilde{c}\right)\right)] \end{array} \end{array}$$

where S is the number of grids (the image is divided into S×S grids to find the position containing lane lines one by one), and B is the number of anchors for each grid to predict the lane line. $I_{ij}^{obj}$ denotes that the jth bounding box predictor in cell *i* is responsible for that prediction; its value is 1 if there is an object in the jth anchor of the ith grid, and otherwise, it is 0. $I_{i}^{obj}$ denotes whether the object (lane line) appears in cell *i*. $\tilde{C_{i}^{j}}$ and $\overset{´}{C_{i}^{j}}$ stand for confidences. $I_{ij}^{noobj}$ denotes that the jth bounding-box predictor in cell *i* is responsible for that prediction. $\lambda_{noobj}$ is a parameter to decrease the loss from confidence predictions for boxes that do not contain lane lines. ${\tilde{p}}_{i}\left(\tilde{c}\right)$ and ${\overset{´}{p}}_{i}\left(\tilde{c}\right)$ represent the probability that the lane line in cell *i* belongs to class $\tilde{c}$, while $\tilde{c}$ is the number of classes (lane-camber category).

## 3. Data Sets’ Descriptions and Detector Model Training

In this section, we present our own data set, elaborate on the training process, and report the evaluation results of the proposed network model.

### 3.1. Data Sets’ Descriptions

**Figure 9:**
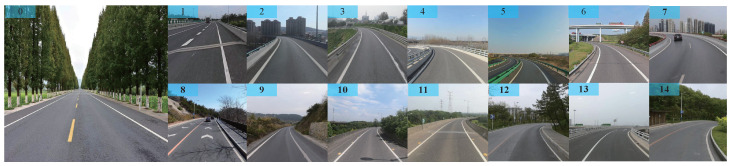
Illustrative images extracted from the acquired unprocessed training data.

The task of creating custom data sets has been previously addressed in our prior research. To collect lane lines and steering angles, a Borgward SUV (BX5) was utilized at 20 km per hour within the Shenyang Qipanshan Scenic Area. Building upon our previous work, we conducted an expansion acquisition experiment at Western Xia Park in Yinchuan. To ensure consistency with the collected steering-angle information from before, we employed the BX5 vehicle once again for experimentation purposes. This time, we recorded 40 h of real-time driving video and augmented the data set by increasing the number of images from its original count of 8000 to 20,000, maintaining the image size of 640 × 640 × 3 pixels. Following statistical analysis on the distribution of collected steering angles, which ranged between −50 and 60 degrees, we divided these angles into 15 categories, ranging from −60 to +60 degrees, as illustrated in Table 3. The label denotes the sequential number of the data sets’ labels. The classification of images in a data set is exemplified in Figure 9. Subsequently, we partitioned our data set into three subsets: a training set (70%), a validation set (20%), and a test set (10%), based on a predetermined ratio.

### 3.2. Measurement Metrics

The performance evaluation of the proposed YOLOv5Ms, based on YOLOv5s, was conducted using precision, recall, mean average precision (mAP@0.5; mAP@0.5:0.95), average detection processing time, parameter count, FLOPs, and model size as measurement metrics [47].

Precision is calculated as the proportion of the number of positive samples correctly predicted to those predicted as positive. It is defined as follows:

(40) $$\begin{array}{c} \begin{array}{cc} \; & Precision=\frac{TP}{TP+FP} \end{array} \end{array}$$

Recall is calculated as the proportion of all targets that are correctly predicted. It is defined as follows:

(41) $$\begin{array}{c} \begin{array}{cc} \; & Recall=\frac{TP}{TP+FN} \end{array} \end{array}$$

The average precision (AP) represents the mean precision rate, which should be maximized while ensuring accuracy as the recall rate gradually increases from 0 to 1. The calculation formulas of mAP@0.5 and mAP@0.5:0.95 are as follows:

(42) $$\begin{array}{c} \begin{array}{cc} \; & mAP=\frac{\sum_{\hat{c}=1}^{\hat{C}}AP\left(\hat{c}\right)}{\hat{C}} \end{array} \end{array}$$

where TP, FP, and FN represent the counts of true positive (TP) cases (indicating correctly predicted lane lines in the model), false positive (FP) cases (denoting situations where no lane line exists but a lane is falsely predicted in the model), and false negative (FN) cases (referring to scenarios where lane lines exist but are incorrectly predicted as absent in the model), respectively. Additionally, $\hat{C}$ denotes the number of curvature categories for lane lines. In this study, we categorized lane-line curves into 15 classes, resulting in $\hat{C}$ being equal to 15. The term mAP@0.5 refers to the mean average precision across all categories when the intersection over union (IoU) is set to 0.5, while mAP@0.5:0.95 represents the average mAP at various IoU thresholds, ranging from 0.5 to 0.95, with a step size of 0.05.

### 3.3. Training and Test Results

Training object detection is a computationally intensive task, particularly following the completion of data-set collection and tagging. In supervised training, the network’s weights or independent parameters are iteratively adjusted to optimize performance for a specific training data set. The proposed model’s training process is executed using the PyTorch framework on the Windows operating system. The software environment includes CUDA 11.0 and Python 3.8. For data-set training, an Advanced Micro Devices (AMD) Ryzen 2700X processor was employed, alongside an NVIDIA GeForce RTX 2080Ti with 11 GB of memory.

**Figure 10:**
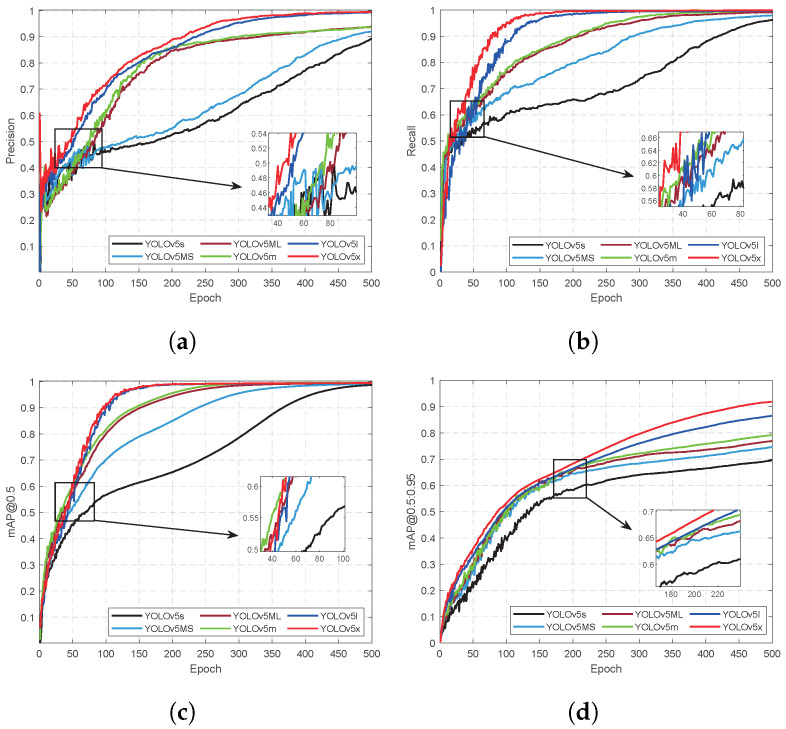
Comparison of the precision, recall rate, mAP@0.5, and mAP@0.5:0.95 of the six models of YOLOv5 in the training and verification stages: (**a**) precision, (**b**) recall, (**c**) mAP@0.5, and (**d**) mAP@0.5:0.95.

**Figure 11:**
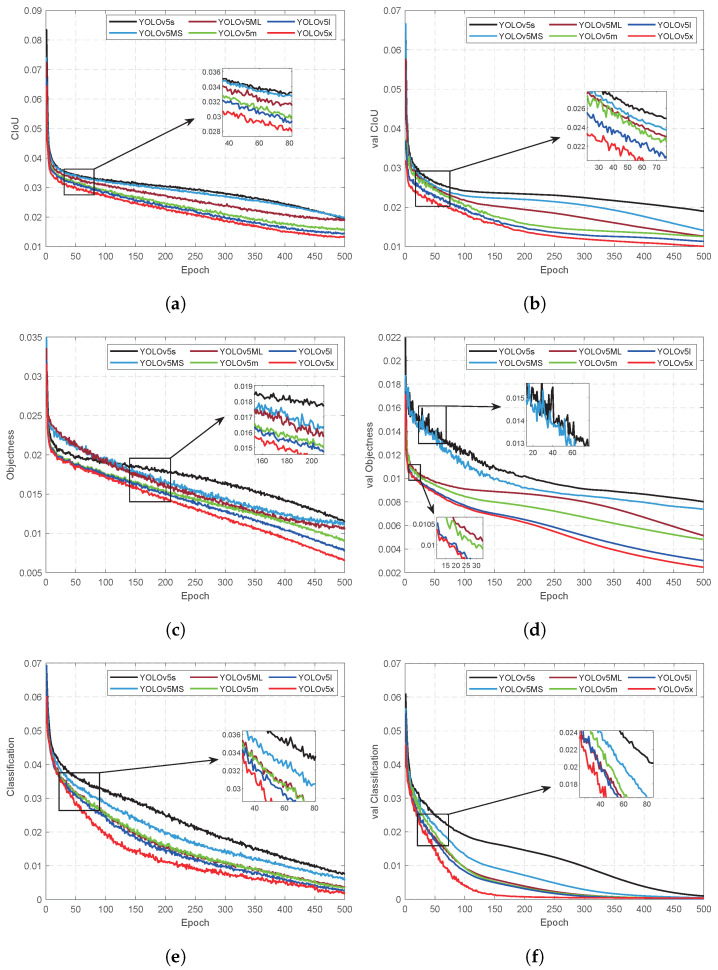
Comparison of the bounding-box regression, confidence, and classification of the six models of YOLOv5 in the training and validation stages: (**a**) CIoU, (**b**) val CIoU, (**c**) objectness, (**d**) val objectness, (**e**) classification, and (**f**) val classification.

Details of the training process and results are shown in Table 4 and Figure 10 and Figure 11. The number of training epochs was set to 500 during network training; hence, ‘Epoch’ in Figure 10 and Figure 11 corresponds to a total of 500 iterations. After the training, the weights of the 6 models were 10.1 M, 36.2 M, 14.5 M, 40.3 M 88.7 M, and 173.3 M, respectively. The total number of parameters of the six models is $5.07\times 10^{6}$, $17.85\times 10^{6}$, $7.05\times 10^{6}$, $20.93\times 10^{6}$, $46.19\times 10^{6}$, and $86.32\times 10^{6}$, respectively. Comparing the training results of our proposed YOLOv5Ms models and the four YOLOv5 models can reveal that the total number of parameters in YOLOv5Ms is reduced by $1.98\times 10^{6}$ compared to YOLOv5s, resulting in a 28.09% decrease and a weight reduction of 4.4 M, which represents a decline of 30.34%. In terms of the training time, under identical hardware conditions, YOLOv5Ms exhibits a training speed that is 5.19 h faster than that of YOLOv5s. Furthermore, regarding precision metrics such as mAP@0.5 and mAP@0.5:0.95, the performance improvement achieved using YOLOv5Ms surpasses that of YOLOv5s, with enhancements reaching 6.59%, 0.41%, and 5.01%, respectively. The experimental results demonstrate that incorporating an attention mechanism into YOLOv5s effectively enhances model precision in reducing both total parameters and weights.

Through a comparison between our proposed YOLOv5Ml model and YOLOv5s and YOLOv5m models, we observed that the various indicators of YOLOv5Ml outperform those of YOLOv5s but are similar to those of YOLOv5m. However, its overall performance is slightly inferior to that of YOLOv5m due to dominant factors such as model size, weight size, and training time. Compared with YOLOv5m, the total number of parameters in our model was reduced by $3.08\times 10^{6}$, the weight size decreased by 4.1 M, and the training time was shortened by 15.72 h.

YOLOv5x and YOLOv5l exhibit superior performance, irrespective of the associated time and hardware costs. However, in practical applications, a common approach is to balance the model cost and overall performance. Therefore, it is generally preferred to consider lower-cost factors such as hardware utilization and execution time while ensuring that the model’s performance meets real-world requirements.

**Figure 12:**
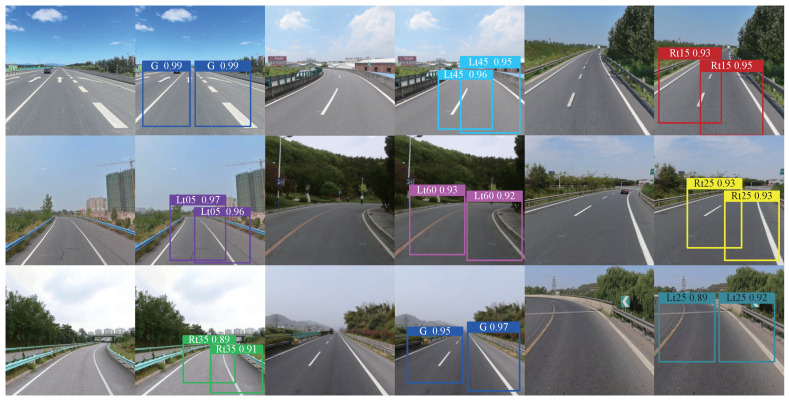
Images labeled with tags and the outcomes of tests.

The test results of the trained model are shown in Figure 12. In Figure 12, the “tags” represent the YOLOv5Ms network subsequent to 500 epochs of training. Following the evaluation of images containing lane lines, the letters denote their respective categories, while the numbers indicate their corresponding probabilities. It can be seen from the test results that the training of the network achieved the expected performance. We discuss the real-time predictive performance of the trained YOLOv5Ms detector by verifying experiments in Section 5.

This paper proposes a composite controller that integrates a novel adaptive updating law and disturbance observer to ensure that AVs’ steering system promptly and accurately responds to the predicted steering-angle signal. Subsequently, we elaborate on the proposed controller in the following section of this paper and conduct a comparative experiment to validate its control performance.

## 4. Mathematical Model of SbW System and Controller Design

### 4.1. Dynamics Model

**Figure 13:**
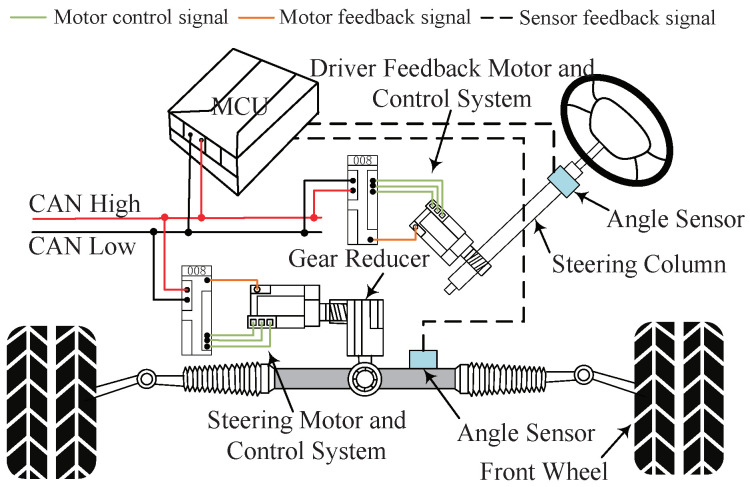
Overall structure diagram of SbW system.

Figure 13 shows the schematic diagram of the SbW system. The SbW system consists mainly of mechanical and electrical structures. It mainly includes a handwheel, a steering column, a steering motor, a motor controller, a gear reducer, an angle sensor, and a feedback motor. On the handwheel side, the angle sensor is mounted on the steering column, and it measures the angle of the handwheel. The motor controller receives the angle signal through CAN to control the motor rotation, and the motor rotation drives the front-wheel rotation through the gear reducer. The sensors mounted on the steering-tie rods measure the steering angle of the front wheels and feedback to the motor controller. The motor controller receives the feedback signal for closed-loop control to track the reference angle. For simplicity, the modeling of the steering system consists of the steering motor and the front wheel, and the dynamic equations of the steering motor are as follows:

(43) $$J_{sm}{\ddot{\delta }}_{s}+B_{sm}{\dot{\delta }}_{s}+\tau_{1}+\tau_{d}=\tau_{m}$$

where $\delta_{s}$ is the angle of the steering motor shaft. $J_{sm}$ is the inertia of the steering motor. $B_{sm}$ is the viscous friction of the steering motor. $\tau_{1}$ represents the torque applied via the front wheels to the steering motor shaft. $\tau_{d}$ is the disturbance torque. $\tau_{m}$ is the torque output via the steering motor.

The dynamic equation for the front wheels is as follows:

(44) $$J_{fw}{\ddot{\delta }}_{f}+B_{fw}{\dot{\delta }}_{f}+\tau_{f}+\tau_{e}=\tau_{2}$$

where $\delta_{f}$ is the angle of the steering motor shaft. $J_{fw}$ is the inertia of the front wheels. $B_{fw}$ is the viscous friction of the steering motor. $\tau_{f}$ is the friction torque on the front wheels. $\tau_{e}$ is the self-aligning torque on the front wheels. $\tau_{2}$ represents the torque transmitted via the steering motor to the front-wheel steering arms. The self-aligning moment is expressed as a hyperbolic tangent function:

(45) $$\tau_{e}=\varrho \tanh\left(\delta_{f}\right)$$

where ϱ is the positive constant related to the road adhesion.

The friction model friction torque is defined as follows [48]:

(46) $$\begin{array}{cc} \tau_{f}= & \alpha_{1}\left[\tanh\left(\beta_{1}{\dot{\delta }}_{f}\right)-\tanh\left(\beta_{2}{\dot{\delta }}_{f}\right)\right]+\alpha_{2}\tanh\left(\beta_{3}{\dot{\delta }}_{f}\right)+\alpha_{3}{\dot{\delta }}_{f} \end{array}$$

where $\alpha_{i}$ and $\beta_{i}$ are positive constants. According to (46), the friction model contains many features of other friction models. Therefore, the friction model (46) provides a more comprehensive description of friction and provides a more accurate model for controller design.

Regardless of the backlash exiting the rack and pinion gear teeth, we have the following relationship:

(47) $$\frac{\delta_{f}}{\delta_{s}}=\frac{{\dot{\delta }}_{f}}{{\dot{\delta }}_{s}}=\frac{{\ddot{\delta }}_{f}}{{\ddot{\delta }}_{s}}=\frac{1}{i_{mc}}=\frac{\tau_{1}}{\tau_{2}}$$

where $i_{mc}$ is the steering ratio.

Using (47) and eliminating $\delta_{s}$, we obtain the following:

(48) $$J_{e}{\ddot{\delta }}_{f}+B_{e}{\dot{\delta }}_{f}+i_{mc}\tau_{d}+\tau_{f}+\tau_{e}=i_{mc}\tau_{m}$$

where $J_{e}=J_{fw}+i_{mc}^{2}J_{sm}$ is the moment of inertia. $B_{e}=B_{fw}+i_{mc}^{2}B_{sm}$ is the the system damping.

We rewrite (48) as follows:

(49) $${\ddot{\delta }}_{f}=\frac{i_{mc}}{J_{e}}\tau_{m}-\frac{B_{e}{\dot{\delta }}_{f}+\tau_{e}+\tau_{f}}{J_{e}}-\frac{i_{mc}\tau_{d}}{J_{e}}$$

We define $x_{1}=\delta_{f}$, $x_{2}={\dot{\delta }}_{f}$ and rewrite (49) as the state space equation as follows:

(50) $$\left\{\begin{array}{c} {\dot{x}}_{1}=x_{2}\\ {\dot{x}}_{2}=i_{e}\tau_{m}-\tau_{ef}-\tau_{D} \end{array}\right.$$

where $i_{e}=\frac{i_{mc}}{J_{e}}$. $\tau_{ef}=\frac{B_{e}{\dot{\delta }}_{f}+\tau_{e}+\tau_{f}}{J_{e}}$ is the lumped uncertainty. $\tau_{D}=\frac{i_{mc}\tau_{d}}{J_{e}}$ is the external disturbance. The design of the controller requires accurate model parameters such as $J_{fw}$, $J_{sm}$ and $i_{mc}$. In engineering practice, these parameters can be obtained through offline parameter identification methods such as the half-period integration method [49] and the zero-mean sinusoidal perturbation method [50]. The transmission ratio $i_{mc}$ can be calculated from the number of gear teeth. However, the accurate friction model $\tau_{f}$ and self-aligning torque $\tau_{e}$ present difficulty in obtaining accurate values. The coefficients $\alpha_{i}$ and $\beta_{i}$ of the friction model are related to road adhesion, and it is difficult to obtain accurate values. The self-aligning torque, $\tau_{e}$, is an estimated model, and the coefficient ρ is related to road adhesion. Therefore, the friction model and the self-aligning torque need to be approximated using other methods in the controller design.

**Lemma 1**  ([51])**.** *For any $\left(x,y\right)\in R^{2}$, the following Young’s inequality is satisfied:*

(51) $$xy\leq \frac{\varpi^{p}}{p}{\left|x\right|}^{p}+\frac{1}{q\varpi^{q}}{\left|y\right|}^{q}$$

*where ϖ>0, p>1, and q>1 are constants satifying that (q−1)(p−1)=1.*

**Lemma 2**  ([52])**.** *A function, F(X): $\Omega_{X}\to R$, with a compact set, $\Omega_{X}\in R^{m}$, can be approximated using a radial basis function neural network (RBFNN):*

(52) $$F\left(X\right)=W^{*T}G\left(X\right)+\varepsilon^{*}$$

*where $W^{*}={\left[W_{1}^{*},W_{2}^{*},...,W_{l}^{*}\right]}^{T}\in R^{l}$ is the ideal weight vector. $X={\left[X_{1},X_{2},...,X_{m}\right]}^{T}\in R^{m}$ is the input vector. $\varepsilon^{*}$ is an optimal approximation. There exists a positive constant, ${\bar{\varepsilon }}_{1}$, that satisfies $\varepsilon^{*}<{\bar{\varepsilon }}_{1}$. $G\left(X\right)=[G_{1}\left(X\right)$,$G_{2}\left(X\right),...,G_{l}\left(X\right)]\in R^{l}$ is the basis function vector. The Gaussian function is chosen as the basis function as follows.*

(53) $$G_{j}\left(X\right)=\exp\left(-\frac{∥X-c_{j}{∥}^{2}}{2b_{j}^{2}}\right)$$

*where $c_{j}={\left[c_{1j},c_{1j},...,c_{mj}\right]}^{T}$ is the center, and $b_{j}$ is the width of the Gaussian function.*

**Remark 1.**  *The disturbance $\tau_{D}$ satisfies the following conditions: $\tau_{D}\leq \tau_{D\max}$ and ${\dot{\tau }}_{D}\leq \varepsilon_{D}$ ($\tau_{D\max}$; $\varepsilon_{D}$∈$R^{+}$). In much of the previous literature [53], it has been assumed that the perturbations and the derivatives of the perturbations are bounded. In this paper, the external disturbances $\tau_{D}=\frac{i_{mc}\tau_{d}}{J_{e}}$, $i_{mc}$ and $J_{e}$ are intrinsic parameters of the system, which are related only to the system itself and not to the external world. In engineering practice, $\tau_{d}$ is mainly an external disturbance including the following: internal disturbances in the steering motor, sensor measurement noise,  external forces acting on the SbW system, and so on. These disturbances and their derivatives exist in practice with unknown maximum values. Therefore, the unknown $\tau_{D\max}$ and $\varepsilon_{D}$ exist such that $\tau_{D}\leq \tau_{D\max}$ and ${\dot{\tau }}_{D}\leq \varepsilon_{D}$.*

### 4.2. Observer Design

Given that the angular position can be measured directly by the sensor in practice, the angular velocity needs to be measured with an additional sensor, and it is difficult to find a suitable location for the sensor. In observer design, the parameters in the state space equations need to be known. However, model uncertainty and external disturbance make it difficult to obtain accurate values. According to Lemma 2, $\tau_{ef}$ can be approximated via RBFNN as follows:

(54) $$\tau_{ef}\left(x_{1},{\hat{x}}_{2}\right)=W_{0}^{*T}G_{0}\left(x_{1},{\hat{x}}_{2}\right)+\varepsilon_{0}^{*}$$

where $W_{0}^{*}$ is the ideal weight. ${\hat{x}}_{2}$ is the angular velocity obtained by the state observer designed later.

Define the estimate of $\tau_{ef}$ as follows:

(55) $${\hat{\tau }}_{ef}={\hat{W}}_{0}G_{0}\left(x_{1},{\hat{x}}_{2}\right)$$

Define $\Delta \tau_{ef}=\tau_{ef}\left(x_{1},x_{2}\right)-\tau_{ef}\left(x_{1},{\hat{x}}_{2}\right)$, and satisfy $|\Delta \tau_{ef}|\leq h_{f}\left|x_{2}-{\hat{x}}_{2}\right|$; $h_{f}$ is a designed positive constant. Therefore, the dynamic of $x_{2}$ can be represented as follows:

(56) $${\dot{x}}_{2}=i_{e}\tau_{m}-\Delta \tau_{ef}-W_{0}^{*T}G_{0}\left(x_{1},{\hat{x}}_{2}\right)+\varepsilon_{0}^{*}-\tau_{D}$$

Define ${\hat{x}}_{1}$ as the estimate of the angular position, ${\hat{x}}_{2}$ as the estimate of angular velocity, ${\tilde{x}}_{1}=x_{1}-{\hat{x}}_{1}$ as the angular position estimation error, and ${\tilde{x}}_{2}=x_{2}-{\hat{x}}_{2}$ as the angular velocity estimation error. Inspired by [54], the state observer design is as follows:

(57) $$\left\{\begin{array}{c} {\dot{\hat{x}}}_{1}={\hat{x}}_{2}+k_{1}{\tilde{x}}_{1}\\ {\hat{x}}_{2}=S_{1}+k_{3}{\tilde{x}}_{1}\\ {\dot{S}}_{1}=i_{e}\tau_{m}-{\hat{W}}_{0}G_{0}\left(x_{1},{\hat{x}}_{2}\right)-{\hat{\tau }}_{D}+k_{2}{\tilde{x}}_{1} \end{array}\right.$$

where $k_{i}\left(i=1,2,3\right)$ is the designed positive constants. ${\hat{\tau }}_{D}$ is the estimate of $\tau_{D}$. According to (50), (56), and (57), the following error dynamics equation is obtained:

(58) $$\left\{\begin{array}{cc} {\dot{\tilde{x}}}_{1}= & -k_{1}{\tilde{x}}_{1}+{\tilde{x}}_{2}\\ {\dot{\tilde{x}}}_{2}= & -\Delta \tau_{ef}-{\tilde{W}}_{0}G_{0}\left(x_{1},{\hat{x}}_{2}\right)-{\tilde{\tau }}_{D}+{\tilde{x}}_{1}\left(k_{1}k_{3}-k_{2}\right)\\ \; & -k_{3}{\tilde{x}}_{2} \end{array}\right.$$

where ${\tilde{\tau }}_{D}=\tau_{D}-{\hat{\tau }}_{D}$ is the disturbance estimation error.

To estimate the disturbance, the auxiliary variable λ is introduced as follows:

(59) $$\lambda =\tau_{D}+\alpha x_{2}$$

where α is a designed constant. The disturbance is estimated as follows:

(60) $${\hat{\tau }}_{D}=\hat{\lambda }-\alpha {\hat{x}}_{2}$$

Deriving λ with respect to time gives the following:

(61) $$\begin{array}{c} \dot{\lambda }={\dot{\tau }}_{D}+\alpha \left(i_{e}\tau_{m}-\tau_{ef}-\lambda +\alpha x_{2}\right) \end{array}$$

The design of $\dot{\hat{\lambda }}$ is as follows:

(62) $$\dot{\hat{\lambda }}=\alpha \left(i_{e}\tau_{m}-\hat{\lambda }+\alpha {\hat{x}}_{2}\right)$$

Define $\tilde{\lambda }=\lambda -\hat{\lambda }$ and combine it with (61) and (62) with the derivation for time to obtain the following:

(63) $$\begin{array}{cc} \dot{\tilde{\lambda }}= & \dot{\lambda }-\dot{\hat{\lambda }}\\ = & {\dot{\tau }}_{D}+\alpha \left(-W_{0}^{*}G_{0}\left(x_{1},{\hat{x}}_{2}\right)+\varepsilon_{0}^{*}+\Delta \tau_{ef}-\tilde{\lambda }+\alpha {\tilde{x}}_{2}\right) \end{array}$$

The Lyapunov function is selected as follows:

(64) $$V_{0}=\frac{1}{2}{\tilde{x}}_{1}^{2}+\frac{1}{2}{\tilde{x}}_{2}^{2}+\frac{1}{2}\tilde{\lambda }$$

Taking the derivative of (64) yields the following:

(65) $$\begin{array}{cc} {\dot{V}}_{0}= & {\tilde{x}}_{1}{\dot{\tilde{x}}}_{1}+{\tilde{x}}_{2}{\dot{\tilde{x}}}_{2}+\tilde{\lambda }\dot{\tilde{\lambda }}\\ = & {\tilde{x}}_{1}{\tilde{x}}_{2}-k_{1}x_{1}^{2}+{\tilde{x}}_{2}\Delta \tau_{ef}-{\tilde{x}}_{2}{\tilde{W}}_{0}^{T}G_{0}\left(x_{1},{\hat{x}}_{2}\right)+{\tilde{x}}_{2}\varepsilon_{0}^{*}\\ & -{\tilde{x}}_{2}{\tilde{\tau }}_{D}+{\tilde{x}}_{1}{\tilde{x}}_{2}\left(k_{1}k_{3}-k_{2}\right)-k_{3}{\tilde{x}}_{2}^{2}+\tilde{\lambda }{\dot{\tau }}_{D}+\alpha \tilde{\lambda }\varepsilon_{0}^{*}\\ \; & +\alpha \tilde{\lambda }\Delta \tau_{ef}-\alpha {\tilde{\lambda }}^{2}+\alpha^{2}\tilde{\lambda }{\tilde{x}}_{2}-\alpha \tilde{\lambda }W_{0}^{*T}G_{0}\left(x_{1},{\hat{x}}_{2}\right) \end{array}$$

Using Lemma 1 yields the following:

(66) $$\left\{\begin{array}{c} {\tilde{x}}_{1}{\tilde{x}}_{2}\leq \frac{\mu_{0}}{2}{\tilde{x}}_{1}^{2}+\frac{1}{2\mu_{0}}{\tilde{x}}_{2}^{2}\\ {\tilde{x}}_{2}\Delta \tau_{ef}\leq \left|h_{f}\right|{\tilde{x}}_{2}^{2}\\ {\tilde{x}}_{2}{\tilde{W}}_{0}^{T}G_{0}\left(x_{1},{\hat{x}}_{2}\right)\leq \frac{\mu_{2}}{2}{\tilde{x}}_{2}^{2}+\frac{1}{2\mu_{1}}{\tilde{W}}_{0}^{T}{\tilde{W}}_{0}\\ {\tilde{x}}_{2}{\tilde{\tau }}_{D}\leq \left(\frac{\mu_{2}}{2}+\frac{\alpha^{2}}{\mu_{2}}\right)x_{2}^{2}+\frac{1}{\mu_{2}}{\tilde{\lambda }}^{2}\\ \tilde{\lambda }{\dot{\tau }}_{D}\leq \frac{\mu_{3}}{2}{\tilde{\lambda }}^{2}+\frac{1}{2\mu_{3}}\varepsilon_{D}^{2}\\ \alpha \tilde{\lambda }W_{0}^{*T}G_{0}\left(x_{1},{\hat{x}}_{2}\right)\leq \frac{\mu_{4}}{2}{\tilde{\lambda }}^{2}+\frac{1}{2\mu_{4}}\alpha^{2}W_{0}^{*T}W_{0}^{*}\\ \alpha^{2}\tilde{\lambda }{\tilde{x}}_{2}\leq \frac{\mu_{5}}{2}{\tilde{\lambda }}^{2}+\frac{1}{2\mu_{5}}\alpha^{4}{\tilde{x}}_{2}^{2}\\ \alpha \tilde{\lambda }\Delta \tau_{ef}\leq \frac{\mu_{6}}{2}{\tilde{\lambda }}^{2}+\frac{1}{2\mu_{6}}\alpha^{2}h_{f}^{2}{\tilde{x}}_{2}^{2}\\ \alpha \tilde{\lambda }\varepsilon_{0}^{*}\leq \frac{\mu_{7}}{2}{\tilde{\lambda }}^{2}+\frac{\alpha^{2}}{2\mu_{7}}\varepsilon_{0}^{*2}\\ {\tilde{x}}_{2}\varepsilon_{0}^{*}\leq \frac{\mu_{8}}{2}{\tilde{x}}_{2}^{2}+\frac{1}{2\mu_{8}}\varepsilon_{0}^{*2} \end{array}\right.$$

According to the above inequalities, we can get the following:

(67) $$\begin{array}{cc} {\dot{V}}_{0}\leq & -\left(k_{1}-\frac{\mu_{0}\left(k_{1}k_{3}-k_{2}+1\right)}{2}\right){\tilde{x}}_{1}^{2}-[k_{3}-\frac{k_{1}k_{3}-k_{2}+1}{2\mu_{0}}\\ \; & +\frac{\mu_{8}}{2}-\frac{\mu_{1}}{2}-\left(\frac{\mu_{2}}{2}+\frac{\alpha^{2}}{\mu_{2}}\right)-\frac{1}{2\mu_{5}}\alpha^{4}-\frac{1}{2\mu_{6}}\alpha^{2}h_{f}^{2}]{\tilde{x}}_{2}^{2}\\ \; & -\left(\alpha -\frac{1}{\mu_{2}}-\frac{\mu_{3}}{2}-\frac{\mu_{4}}{2}-\frac{\mu_{5}}{2}-\frac{\mu_{6}}{2}-\frac{\mu_{7}}{2}\right){\tilde{\lambda }}^{2}+\frac{1}{2\mu_{3}}\varepsilon_{D}^{2}\\ \; & +\frac{1}{2\mu_{1}}{\tilde{W}}_{0}^{T}{\tilde{W}}_{0}+\frac{1}{2\mu_{4}}\alpha^{2}W_{0}^{*T}W_{0}^{*}+\left(\frac{\alpha^{2}}{2\mu_{7}}+\frac{1}{2\mu_{8}}\right)\varepsilon_{0}^{*2} \end{array}$$

where $\kappa_{1}=k_{1}-\frac{\mu_{0}\left(k_{1}k_{3}-k_{2}+1\right)}{2}$, $\kappa_{2}=k_{3}-\frac{k_{1}k_{3}-k_{2}+1}{2\mu_{0}}+\frac{\mu_{8}}{2}-\frac{\mu_{1}}{2}-\left(\frac{\mu_{2}}{2}+\frac{\alpha^{2}}{\mu_{2}}\right)-\frac{1}{2\mu_{5}}\alpha^{4}-\frac{1}{2\mu_{6}}\alpha^{2}h_{f}^{2}$, $\kappa_{3}=\alpha -\frac{1}{\mu_{2}}-\frac{\mu_{3}}{2}-\frac{\mu_{4}}{2}-\frac{\mu_{5}}{2}-\frac{\mu_{6}}{2}-\frac{\mu_{7}}{2}$, $\kappa_{4}=\frac{1}{2\mu_{3}}\varepsilon_{D}^{2}+\frac{1}{2\mu_{1}}{\tilde{W}}_{0}^{T}{\tilde{W}}_{0}+\frac{1}{2\mu_{4}}\alpha^{2}W_{0}^{*T}W_{0}^{*}+\left(\frac{\alpha^{2}}{2\mu_{7}}+\frac{1}{2\mu_{8}}\right)\varepsilon_{0}^{*2}$.

According to (67), if $\kappa_{1}>0$, $\kappa_{2}>0$, $\kappa_{3}>0$, and $\kappa_{4}$ is bounded, it follows from Lyapunov’s stability theory that the estimation errors ${\tilde{x}}_{1}$, ${\tilde{x}}_{2}$, and $\tilde{\lambda }$ are ultimately and consistently bounded. Therefore, this paper aims to design a suitable adaptive control method to guarantee that all states of the closed-loop system, including the observer, are bounded.

### 4.3. Controller Design

In order to improve the tracking accuracy, a barrier Lyapunov function is used on the basis of the traditional backstepping control method. The proposed controller design is divided into two steps.

Step 1: Define the variables $z_{1}=x_{1}-x_{d}$ and $z_{2}={\hat{x}}_{2}-\alpha_{1}$. Select the barrier Lyapunov function as follows:

(68) $$V_{1}=\frac{1}{2}\ln\frac{z_{1}^{2}}{b_{1}^{2}-z_{1}^{2}}$$

where $b_{1}$ is a designed positive constant for the boundary.

The derivation of (68) yields the following:

(69) $${\dot{V}}_{1}=\frac{z_{1}{\dot{z}}_{1}}{b_{1}^{2}-z_{1}^{2}}=\frac{z_{1}}{b_{1}^{2}-z_{1}^{2}}\left(z_{2}+\alpha_{1}+{\tilde{x}}_{2}-{\dot{x}}_{d}\right)$$

The virtual control law $\alpha_{1}$ is designed as follows:

(70) $$\alpha_{1}=-k_{d1}z_{1}+{\dot{x}}_{d}-\frac{z_{1}}{b_{1}^{2}-z_{1}^{2}}-\frac{5}{2}\left(b_{1}^{2}-z_{1}^{2}\right)z_{1}$$

Substituting (70) into (69) yields the following:

(71) $$\begin{array}{cc} {\dot{V}}_{1}= & \frac{z_{1}}{b_{1}^{2}-z_{1}^{2}}(z_{2}-k_{d1}z_{1}+{\dot{x}}_{d}-\frac{z_{1}}{b_{1}^{2}-z_{1}^{2}}-\frac{5}{2}\left(b_{1}^{2}-z_{1}^{2}\right)\\ \; & -{\dot{x}}_{d}+{\tilde{x}}_{2})\\ = & \frac{z_{1}z_{2}}{b_{1}^{2}-z_{1}^{2}}-\frac{k_{d1}z_{1}^{2}}{b_{1}^{2}-z_{1}^{2}}-\frac{z_{1}^{2}}{{\left(b_{1}^{2}-z_{1}^{2}\right)}^{2}}-\frac{5}{2}z_{1}^{2}+\frac{z_{1}{\tilde{x}}_{2}}{b_{1}^{2}-z_{1}^{2}} \end{array}$$

Using Lemma 1 yields the following:

(72) $$\left\{\begin{array}{c} \frac{z_{1}z_{2}}{b_{1}^{2}-z_{1}^{2}}\leq \frac{1}{2}\frac{z_{1}^{2}}{{\left(b_{1}^{2}-z_{1}^{2}\right)}^{2}}+\frac{1}{2}z_{2}^{2}\\ \frac{z_{1}{\tilde{x}}_{2}}{b_{1}^{2}-z_{1}^{2}}\leq \frac{1}{2}\frac{z_{1}^{2}}{{\left(b_{1}^{2}-z_{1}^{2}\right)}^{2}}+\frac{1}{2}{\tilde{x}}_{2}^{2} \end{array}\right.$$

Substituting (72) into (71) yields the following:

(73) $${\dot{V}}_{1}\leq -\frac{k_{d1}z_{1}^{2}}{b_{1}^{2}-z_{1}^{2}}+\frac{1}{2}z_{2}^{2}-\frac{5}{2}z_{1}^{2}+\frac{1}{2}{\tilde{x}}_{2}^{2}$$

Step 2: Select the barrier Lyapunov function as follows:

(74) $$V_{2}=\frac{1}{2}\ln\frac{z_{2}^{2}}{b_{2}^{2}-z_{2}^{2}}+\frac{1}{2r_{0}}{\tilde{W}}_{0}^{T}{\tilde{W}}_{0}+\frac{1}{2r_{1}}{\tilde{W}}_{1}^{T}{\tilde{W}}_{1}$$

where $b_{2}$ is a designed positive constant for the boundary. $r_{0}$ and $r_{1}$ are positive constants.

The derivation of (74) yields the following:

(75) $$\begin{array}{cc} {\dot{V}}_{2}= & \frac{z_{2}{\dot{z}}_{2}}{b_{2}^{2}-z_{2}^{2}}-\frac{1}{r_{0}}{\tilde{W}}_{0}^{T}{\dot{W}}_{0}-\frac{1}{r_{0}}{\tilde{W}}_{1}^{T}{\dot{W}}_{1}\\ = & \frac{z_{2}}{b_{2}^{2}-z_{2}^{2}}(i_{e}\tau_{m}-{\hat{\tau }}_{ef}-{\hat{\tau }}_{D}+k_{3}{\tilde{x}}_{2}+\left(k_{2}-k_{1}k_{3}\right){\tilde{x}}_{1}\\ \; & -{\dot{\alpha }}_{1})-\frac{1}{r_{0}}{\tilde{W}}_{0}^{T}{\dot{W}}_{0}-\frac{1}{r_{1}}{\tilde{W}}_{1}^{T}{\dot{W}}_{1} \end{array}$$

Define $F={\dot{\alpha }}_{1}$; according to Lemma 2, *F* can be approximated via RBFNN as follows:

(76) $$F=W_{1}^{*T}G_{1}\left(x_{1},{\hat{x}}_{2}\right)+\varepsilon_{1}^{*}$$

Define the estimate of *F* as follows:

(77) $$\hat{F}={\hat{W}}_{1}G_{1}\left(x_{1},{\hat{x}}_{2}\right)$$

The control input *u* is designed as follows:

(78) $$\begin{array}{cc} \tau_{m}= & \frac{1}{i_{e}}[{\hat{\tau }}_{ef}+{\hat{\tau }}_{D}-\left(k_{2}-k_{1}k_{3}\right){\tilde{x}}_{1}-k_{d2}z_{2}-\frac{k_{3}^{2}z_{2}}{b_{2}^{2}-z_{2}^{2}}\\ \; & -\frac{3z_{2}}{2(b_{2}^{2}-z_{2}^{2})}-z_{1}-\frac{5}{2}\left(b_{2}^{2}-z_{2}^{2}\right)z_{2}+\hat{F}] \end{array}$$

The neural network adaptive law is designed as follows:

(79) $$\left\{\begin{array}{c} {\dot{\hat{W}}}_{0}=r_{0}\left(z_{1}+z_{2}\right)G_{0}-m_{0}{\hat{W}}_{0}\\ {\dot{\hat{W}}}_{1}=r_{1}\left(z_{1}+z_{2}\right)G_{1}-m_{1}{\hat{W}}_{1} \end{array}\right.$$

where $r_{i},m_{i}\left(i=1,2\right)$ is the designed positive constants.

Substituting (77)–(79) into (75) yields the following:

(80) $$\begin{array}{cc} {\dot{V}}_{2}= & \frac{k_{3}z_{2}{\tilde{x}}_{2}}{b_{2}^{2}-z_{2}^{2}}-\frac{z_{2}}{b_{2}^{2}-z_{2}^{2}}{\tilde{W}}_{1}^{T}G_{1}+\frac{z_{2}}{b_{2}^{2}-z_{2}^{2}}\varepsilon_{1}^{*}-\frac{k_{d2}z_{2}^{2}}{b_{2}^{2}-z_{2}^{2}}\\ \; & -\frac{3z_{2}^{2}}{2{\left(b_{2}^{2}-z_{2}^{2}\right)}^{2}}-\frac{k_{3}^{2}z_{2}^{2}}{2{\left(b_{2}^{2}-z_{2}^{2}\right)}^{2}}-\frac{z_{1}z_{2}}{b_{2}^{2}-z_{2}^{2}}-\frac{5}{2}z_{2}^{2}\\ \; & -{\tilde{W}}_{0}^{T}\left(z_{1}+z_{2}\right)G_{0}+\frac{m_{0}}{r_{0}}{\hat{W}}_{0}-{\tilde{W}}_{1}^{T}\left(z_{1}+z_{2}\right)G_{1}+\frac{m_{1}}{r_{1}}{\hat{W}}_{1} \end{array}$$

Using Lemma 1 yields the following:

(81) $$\left\{\begin{array}{c} \frac{k_{3}z_{2}{\tilde{x}}_{2}}{b_{2}^{2}-z_{2}^{2}}\leq \frac{k_{3}^{2}z_{2}^{2}}{2{\left(b_{2}^{2}-z_{2}^{2}\right)}^{2}}+\frac{1}{2}{\tilde{x}}_{2}^{2}\\ \frac{z_{2}}{b_{2}^{2}-z_{2}^{2}}{\tilde{W}}_{1}G_{1}\leq \frac{z_{2}^{2}}{2{\left(b_{2}^{2}-z_{2}^{2}\right)}^{2}}+\frac{1}{2}{\tilde{W}}_{1}^{T}{\tilde{W}}_{1}\\ \frac{z_{2}\varepsilon_{1}^{*}}{b_{2}^{2}-z_{2}^{2}}\leq \frac{z_{2}^{2}}{2{\left(b_{2}^{2}-z_{2}^{2}\right)}^{2}}+\frac{1}{2}\varepsilon_{1}^{*2}\\ \frac{z_{1}z_{2}}{b_{2}^{2}-z_{2}^{2}}\leq \frac{z_{2}^{2}}{2{\left(b_{2}^{2}-z_{2}^{2}\right)}^{2}}+\frac{1}{2}z_{1}^{2}\\ -{\tilde{W}}_{0}^{T}\left(z_{1}+z_{2}\right)G_{0}+\frac{m_{0}}{r_{0}}{\hat{W}}_{0}\leq z_{1}^{2}+z_{2}^{2}+\frac{1}{2}{\tilde{W}}_{0}^{T}{\tilde{W}}_{0}\\ -\frac{m_{0}}{2r_{0}}{\tilde{W}}_{0}^{T}{\tilde{W}}_{0}+\frac{m_{0}}{2r_{0}}W_{0}^{*T}W_{0}^{*}\\ -{\tilde{W}}_{1}^{T}\left(z_{1}+z_{2}\right)G_{1}+\frac{m_{1}}{r_{1}}{\hat{W}}_{1}\leq z_{1}^{2}+z_{2}^{2}+\frac{1}{2}{\tilde{W}}_{1}^{T}{\tilde{W}}_{1}\\ -\frac{m_{1}}{2r_{1}}{\tilde{W}}_{1}^{T}{\tilde{W}}_{1}+\frac{m_{1}}{2r_{1}}W_{1}^{*T}W_{1}^{*} \end{array}\right.$$

**Figure 14:**
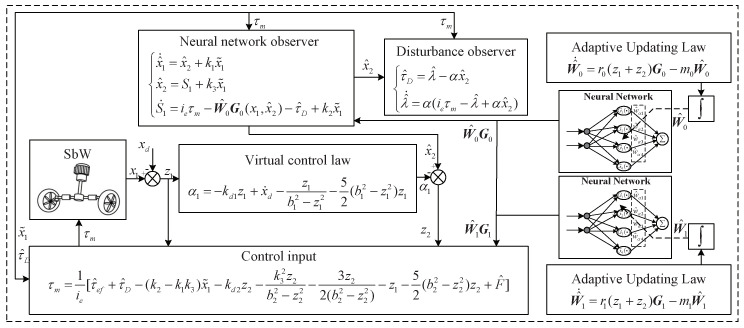
The control principles and procedures of the method proposed in this paper.

The control principles and procedures of the constraint-based adaptive neural network-output feedback controller proposed in this paper is shown in Figure 14.

Substituting (81) into (80) yields the following:

(82) $$\begin{array}{cc} {\dot{V}}_{2}\leq & \frac{1}{2}{\tilde{x}}_{2}^{2}-\frac{k_{d2}z_{2}^{2}}{b_{2}^{2}-z_{2}^{2}}-\left(\frac{m_{0}}{2r_{0}}-\frac{1}{2}\right){\tilde{W}}_{0}^{T}{\tilde{W}}_{0}-\left(\frac{m_{1}}{2r_{1}}-\frac{1}{2}\right){\tilde{W}}_{1}^{T}{\tilde{W}}_{1}\\ \; & +\frac{1}{2}\varepsilon_{1}^{*}-\frac{1}{2}z_{2}^{2}+\frac{5}{2}z_{1}^{2}+\frac{m_{0}}{2r_{0}}W_{0}^{*T}W_{0}^{*}+\frac{m_{1}}{2r_{1}}W_{1}^{*T}W_{1}^{*} \end{array}$$

### 4.4. Stability Analysis

**Theorem 1.**  *For the SbW control system (50), the neural network state observer (57) and disturbance observer (60), the virtual controller (70), and the controller (78) are used. The adaptive law of the neural network is defined in (79); then, all signals of the closed-loop system converge to a compact set.*

**Proof.**  Select the Lyapunov function as follows:

(83) $$V=V_{0}+V_{1}+V_{2}$$

Substituting (67), (73), and (82) into (83) yields the following:

(84) $$\begin{array}{cc} \dot{V}\leq & -\left(k_{1}-\frac{\mu_{0}\left(k_{1}k_{3}-k_{2}+1\right)}{2}\right){\tilde{x}}_{1}^{2}-(k_{3}-\frac{k_{1}k_{3}-k_{2}+1}{2\mu_{0}}\\ \; & +\frac{\mu_{8}}{2}-\frac{\mu_{1}}{2}-\frac{\mu_{2}}{2}-\frac{\alpha^{2}}{\mu_{2}}-\frac{1}{2\mu_{5}}\alpha^{4}-\frac{1}{2\mu_{6}}\alpha^{2}h_{f}^{2}-1){\tilde{x}}_{2}^{2}\\ \; & -\left(\alpha -\frac{1}{\mu_{2}}-\frac{\mu_{3}}{2}-\frac{\mu_{4}}{2}-\frac{\mu_{5}}{2}-\frac{\mu_{6}}{2}-\frac{\mu_{7}}{2}\right){\tilde{\lambda }}^{2}\\ \; & -\left(\frac{m_{0}}{2r_{0}}-\frac{1}{2}-\frac{1}{2\mu_{1}}\right){\tilde{W}}_{0}^{T}{\tilde{W}}_{0}-\left(\frac{m_{1}}{2r_{1}}-\frac{1}{2}\right){\tilde{W}}_{1}^{T}{\tilde{W}}_{1}\\ \; & -k_{d1}\frac{z_{1}^{2}}{b_{1}^{2}-z_{1}^{2}}-k_{d2}\frac{z_{2}^{2}}{b_{2}^{2}-z_{2}^{2}}+\frac{1}{2\mu_{3}}\varepsilon_{D}^{2}+\frac{1}{2\mu_{4}}W_{0}^{*T}W_{0}^{*}\\ \; & +\left(\frac{\alpha^{2}}{2\mu_{7}}+\frac{1}{2\mu_{4}}\right)\varepsilon_{0}^{*2}+\frac{1}{2}\varepsilon_{1}^{*}+\frac{m_{0}}{2r_{0}}W_{0}^{*T}W_{0}^{*}+\frac{m_{1}}{2r_{1}}W_{1}^{*T}W_{1}^{*} \end{array}$$

where $C_{1}=2\left(k_{1}-\frac{\mu_{0}\left(k_{1}k_{3}-k_{2}+1\right)}{2}\right)$, $C_{2}=2\left(k_{3}-\frac{k_{1}k_{3}-k_{2}+1}{2\mu_{0}}+\frac{\mu_{8}}{2}-\frac{\mu_{1}}{2}-\frac{\mu_{2}}{2}-\frac{\alpha^{2}}{\mu_{2}}-\frac{1}{2\mu_{5}}\alpha^{4}-\frac{1}{2\mu_{6}}\alpha^{2}h_{f}^{2}-1\right)$, $C_{3}=2\left(\alpha -\frac{1}{\mu_{2}}-\frac{\mu_{3}}{2}-\frac{\mu_{4}}{2}-\frac{\mu_{5}}{2}-\frac{\mu_{6}}{2}-\frac{\mu_{7}}{2}\right)$, $C_{4}=2k_{d1}$, $C_{5}=2k_{d2}$, $C_{6}=2\left(\frac{m_{0}}{2r_{0}}-\frac{1}{2}-\frac{1}{2\mu_{1}}\right)$, $C_{7}=2\left(\frac{m_{1}}{2r_{1}}-\frac{1}{2}\right)$, $C_{0}=+\frac{1}{2\mu_{3}}\varepsilon_{D}^{2}+\frac{1}{2\mu_{4}}W_{0}^{*T}W_{0}^{*}+\left(\frac{\alpha^{2}}{2\mu_{7}}+\frac{1}{2\mu_{4}}\right)\varepsilon_{0}^{*2}+\frac{1}{2}\varepsilon_{1}^{*}+\frac{m_{0}}{2r_{0}}W_{0}^{*T}W_{0}^{*}+\frac{m_{1}}{2r_{1}}W_{1}^{*T}W_{1}^{*}$.

Define the compact set $\Omega_{z}=\{z_{i}\left|\right|z_{i}\left|\leq b_{i},i=1,2\right\}$; then, on $\Omega_{z}$, the following is obtained: $\ln\frac{z_{i}^{2}}{b_{i}^{2}-z_{i}^{2}}\leq \frac{z_{i}^{2}}{b_{i}^{2}-z_{i}^{2}}$. Therefore, (84) can be expressed as follows:

(85) $$\dot{V}\leq -aV+C_{0}$$

where $a=\min\{C_{1},C_{2},C_{3},C_{4},C_{5},C_{6},C_{7}\}$.

According to A, ${\tilde{x}}_{1}$, ${\tilde{x}}_{2}$, λ, $z_{1}$, $z_{2}$, ${\tilde{W}}_{0}$, and ${\tilde{W}}_{1}$ are bounded. Since α is bounded, it follows that ${\hat{x}}_{2}=z_{2}+\alpha$ is bounded, and $x_{2}={\hat{x}}_{2}+{\tilde{x}}_{2}$ is bounded. It can be inferred from (70) and (78) that the virtual control law and control input are also bounded. Theorem 1 has been proven.    □

## 5. Simulation and Experimental Validation

In this section, to verify the effectiveness of the proposed controller in the online control SbW system, we conducted simulations and hardware loop experiments.

### 5.1. Simulation

The parameters of the SbW system are chosen to be the same with [22], which are listed in Table 5. In this numerical simulation, the parameters are set as follows: $\alpha_{1}=0.25$, $\alpha_{2}=20$, $\alpha_{3}=0.01$, $\beta_{1}=100$, $\beta_{2}=1$, and $\beta_{3}=100$. To verify the robustness under different loads, different external disturbance are added as follows:

(86) $$\tau_{D}=\left\{\begin{array}{c} 2,0<t\leq 40\\ 1+0.5tanh(0.1(t-40)),40<t\leq 80\\ 2+0.3tanh(0.1(t-80)),80<t\leq 120\\ 3-120tanh(0.1(t-120)),120<t\leq 160 \end{array}\right.$$

To verify the superiority of the proposed method, three methods are selected for comparison. In order to ensure a fair comparison of the three methods, the parameters of each method are adjusted to achieve the best control effect under the same conditions.

(1) The controller proposed in Theorem 1 is expressed as follows:

(87) $$\begin{array}{cc} \tau_{m}= & \frac{1}{i_{e}}[{\hat{\tau }}_{ef}+{\hat{\tau }}_{D}-\left(k_{2}-k_{1}k_{3}\right){\tilde{x}}_{1}-k_{d2}z_{2}-\frac{k_{3}^{2}z_{2}}{b_{2}^{2}-z_{2}^{2}}\\ \; & -\frac{3z_{2}}{2(b_{2}^{2}-z_{2}^{2})}-z_{1}-\frac{5}{2}\left(b_{2}^{2}-z_{2}^{2}\right)z_{2}+\hat{F}] \end{array}$$

The parameters of the proposed method are set as follows: $k_{1}=0.001$, $k_{2}=150$, $k_{3}=50$, $r_{0}=100$, $m_{0}=20$, $r_{1}=100$, $m_{1}=15$, $k_{d1}=k_{d2}=10$, $b_{1}=b_{2}=5$, α=1. The neural network parameters are set as follows: $b_{j}=5$, $c_{1}={\left[0,0\right]}^{T}$, $c_{2}={\left[0.05,0.05\right]}^{T}$, $c_{3}={\left[0.1,0.1\right]}^{T}$, $c_{4}={\left[0.15,0.15\right]}^{T}$, $c_{5}={\left[0.2,0.2\right]}^{T}$, $c_{6}={\left[0.25,0.25\right]}^{T}$, $c_{7}={\left[0.3,0.3\right]}^{T}$, $c_{8}={\left[0.35,0.35\right]}^{T}$, $c_{9}={\left[0.4,0.4\right]}^{T}$, $c_{10}={\left[0.45,0.45\right]}^{T}$, $c_{11}={\left[0.5,0.5\right]}^{T}$, $c_{12}={\left[0.55,0.55\right]}^{T}$, $c_{13}={\left[0.6,0.6\right]}^{T}$, $c_{14}={\left[0.65,0.65\right]}^{T}$, $c_{15}={\left[0.7,0.7\right]}^{T}$, $c_{16}={\left[0.75,0.75\right]}^{T}$, $c_{17}={\left[0.8,0.8\right]}^{T}$, $c_{18}={\left[0.85,0.85\right]}^{T}$, $c_{19}={\left[0.9,0.9\right]}^{T}$, $c_{20}={\left[0.95,0.95\right]}^{T}$, $c_{21}={\left[1,1\right]}^{T}$.

(2) To verify the effectiveness of the disturbance observer, this method removes the disturbance observer from the proposed method, and the controller parameters remain consistent with the proposed method.

(3) The traditional backstepping control method (BSC) differs from the proposed method by removing the state constraints and keeping the other parts the same. The controller is expressed as follows:

(88) $$\left\{\begin{array}{c} \alpha_{1}=-k_{d1}z_{1}+{\dot{x}}_{d}\\ \tau_{m}=\frac{1}{i_{e}}\left({\hat{\tau }}_{ef}+{\hat{\tau }}_{D}+{\dot{\alpha }}_{1}-\left(k_{2}-k_{1}k_{3}\right){\tilde{x}}_{1}-k_{d2}z_{2}-z_{1}\right) \end{array}\right.$$

**Figure 15:**
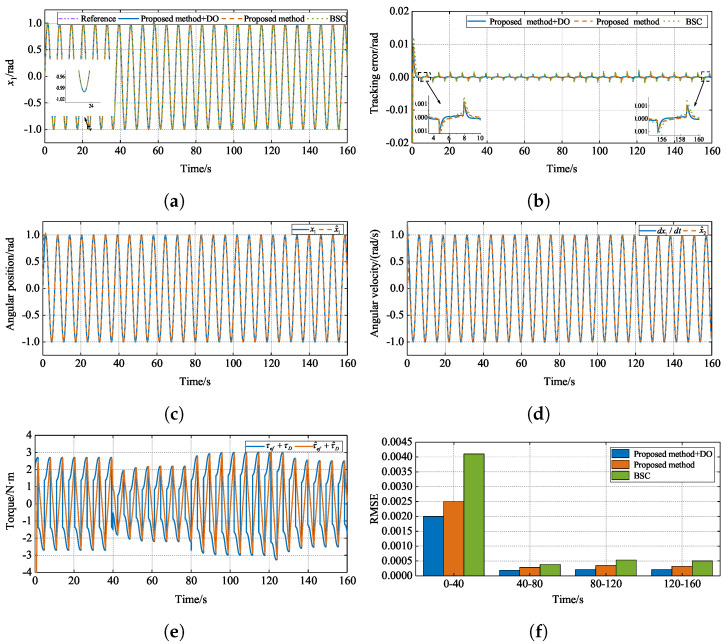
The simulation results. **(a**) Position-tracking performance; (**b**) tracking error; (**c**) angular position; (**d**) angular velocity; (**e**) friction and torque; (**f**) RMSE.

The parameters of BSC are set as follows: $k_{d1}=k_{d2}=70$. The parameters of the state observer and the disturbance observer are consistent with the proposed method. The simulation reference signal is set to $x_{d}=\sin\left(t\right)$. In order to evaluate the performance of the three controllers, a performance index is introduced: the root mean square error (RMSE), RMSE=$\sqrt{\frac{1}{n}\sum_{i=1}^{n}e_{i}^{2}}$. The simulation results are shown in Figure 15.

Figure 15 shows the curves of the numerical simulation. Figure 15a,b show the position-tracking performance and tracking error of the three methods, respectively. From Figure 15a, it can be seen that all three methods can track the reference signal well,  the errors are very close to each other, and it is difficult to see the difference between the three methods intuitively. However, the tracking error curve shows that the tracking error of BSC is more than 0.01 rad in the starting tracking stage, while the other two methods are within 0.01 rad, which indicates that the proposed method achieves a faster response speed. During the tracking process, the tracking error of BSC is larger than that of the other two methods. The tracking error of the proposed method with a disturbance observer is smaller, and the tracking error converges near zero faster. Figure 15c,d show the curves of the estimation results of the state observer for $x_{1}$ and $x_{2}$, respectively. As can be seen from Figure 15c, at the beginning stage, the curves of ${\hat{x}}_{1}$ and $x_{1}$ have a slightly larger error and do not fit perfectly. But soon after that, the two curves almost coincide, and the state observer can estimate the angular position well. As can be seen from Figure 15d, since the simulation is an ideal environment to derive the angular velocity directly for the angular position without noise, a smooth curve is obtained. The curves of ${\hat{x}}_{2}$ and $dx_{1}/dt$ almost coincide, and the state observer can estimate the angular velocity well. Figure 15e shows the curve of the neural network approximation of friction and the disturbance observer estimation of external disturbance; from the simulation results, it can be seen that the neural network and the disturbance observer are quickly adjusted by updating the adaptive law to accurately estimate the friction and the external disturbance when the load changes. Figure 15f shows RSME histograms of the three methods, from which it can be seen that, in each period, the proposed method with the disturbance observer achieved lower values in RMSE, indicating better tracking results.

### 5.2. Experiment

**Figure 16:**
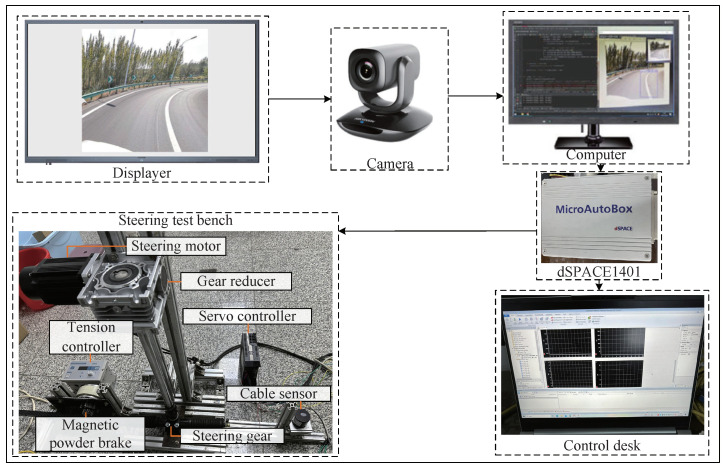
The schematic diagram of the YOLOv5-based end-to-end steering control for an autonomous vehicle.

In order to better validate the effectiveness of the proposed method, and for safety reasons, hardware-in-the-loop (HIL) experiments were conducted to verify the effectiveness of the proposed control method in practice. The schematic diagram of the steering control YOLOv5-based end-to-end for an autonomous vehicle is shown in Figure 16. The SbW system mainly consists of a steering test bench, dSPACE1401, a monocular camera, and a computer. The steering test bench mainly consists of a steering motor, a reducer, a servo controller, a tension controller, a magnetic powder brake, a steering gear, a cable sensor, and a table frame. In order to verify the proposed method’s effectiveness and superiority in comparison with two other methods, firstly, the steering-angle signal received via the controller is artificially given, which causes the steering wheel to traverse the categorized steering angles sequentially, starting from the original position, which ranges between −60° and 60°.

The parameters of the methods in the hardware-in-the-loop experiments are different from those in the simulation, and the parameters of the three methods are set as follows:

(1) The parameters of the proposed method are set to $k_{1}=0.001$, $k_{2}=180$, $k_{3}=100$, $k_{d1}=70$, $k_{d2}=1$, $b_{1}=0.2$, $b_{2}=0.3$, and α=0.7. The parameters of the neural network are consistent with those in the simulation.

(2) The parameters of BSC are set to $k_{d1}=85$, $k_{d2}=2$. The other parameters are consistent with the proposed method.

**Figure 17:**
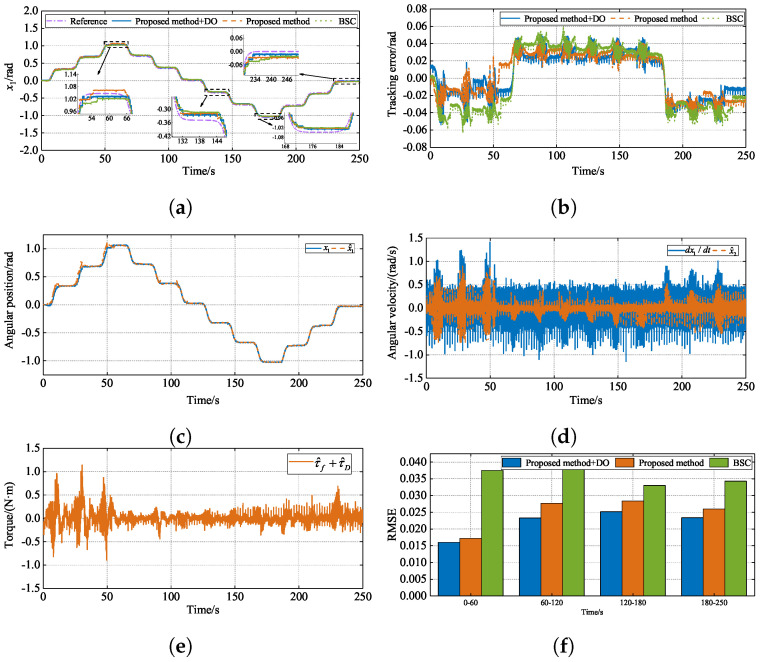
The experimental results. (**a**) Position-tracking performance; (**b**) tracking error; (**c**) angular position; (**d**) angular velocity; (**e**) friction and torque; (**f**) RMSE.

The experimental results are shown in Figure 17. Figure 17a,b show the position-tracking performance and tracking error, respectively. From Figure 17a, it can be seen that the tracking performance curve is not as smooth as in the simulation due to the noise in the angular position of the sensor measurement in the experiment. Compared to the other two methods, the proposed method with the disturbance observer exhibits a faster response and a smaller steady-state error. This is more intuitive in the tracking error curve. As Figure 17b shows, the maximum tracking error of the BSC is 0.055 rad, and the maximum tracking errors of the other two methods are within 0.055 rad. Within 50 s to 120 s, it is clearly seen that the proposed method with perturbation observer has a smaller tracking error. In the final stage of tracking, the proposed method with the perturbed observer has a smaller steady-state error, and the tracking error is closer to zero. Figure 17c,d show the estimated curves for the angular position and angular velocity, respectively. From Figure 17c, it can be seen that the ${\hat{x}}_{1}$ curve derived from the state observer is very close to that of $x_{1}$. Between 0 and 50 s, due to the initial operation of the SbW system, there is significant resistance between various components, such as the gap between the gear and the rack, and the gap between the steering motor and the gear reducer. After running for a period of time, the system operates in normal working condition. This can also be seen in Figure 17e, where the estimation of friction and external disturbances from 0 to 50 s is larger than after 50 s. Therefore, the estimation of the angle position has a certain impact, and the error from 0 to 50 s is larger than that after 50 s. From Figure 17d, it can be seen that the derivation of $x_{1}$ leads to an amplification in the noise due to the presence of noise in the sensor measurements of the experiment. The ${\hat{x}}_{2}$ derived from the state observer, on the other hand, is much closer to the real value of $x_{2}$, has much less noise, and meets the requirements of the controller design. Figure 17e shows the neural network approximation friction and the external disturbance curve estimated via the disturbance observer. Figure 17f shows the RMSE curves of the three methods; at each stage, the proposed method has smaller values, indicating that the proposed method with the disturbance observer achieves better tracking performance.

From the above simulation and experiment, it can be seen that, although the experimental conditions added in the simulation are different from the environment in the actual experiment, it is difficult to achieve consistency with the simulation in the actual experimental environment; for instance, noise is present in the sensors, the actuator has a response delay, added friction occurs, and the external perturbation is inconsistent, etc. However, the trend in the results obtained from the numerical simulation and HIL experiment is consistent, indicating the superiority of the method proposed in this paper.

**Figure 18:**
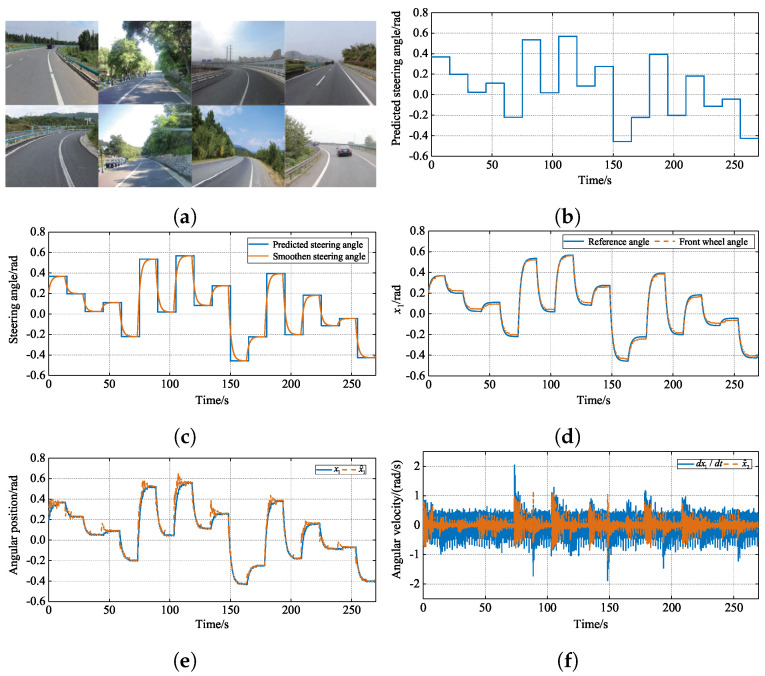
The real-time prediction and execution of the steering angle. (**a**) Randomly selected images; (**b**) predicted angle; (**c**) reference angle; (**d**) position tracking performance; (**e**) angular position; (**f**) angular velocity.

For safety reasons, we verified the real-time steering prediction and steering control of the trained YOLOv5 on the SbW experimental bench. The experimental results are shown in Figure 18. Figure 18a shows the randomly selected real-time validation images; the trained YOLOv5 outputs the predicted steering angle based on the image and sends it to follow the controller via a serial protocol. Figure 18b shows the output predicted steering angle. When the predicted angle changes, the reference signal is a step-signal change. While, in practice, there are delays in the communication between the steering motor and the controller, it is difficult to track the step signal, and the actual tracking performance is fully and smoothly shifted. Therefore, a filter is added after the predicted steering angle of the output to obtain a smoothed reference signal, as shown in Figure 18c. The adaptive neural network output feedback controller proposed in this paper is used to track the reference signal derived earlier, as shown in Figure 18d, from which it can be seen that, in real-time turn angle variation, the method proposed in this paper can still track the reference angle well and achieves good tracking performance. Figure 18e,f show the estimation curves of the state observer, from which it can be seen that ${\hat{x}}_{1}$ and ${\hat{x}}_{2}$ derived from the state observer achieve good accuracy when the reference turning angle varies randomly, which is consistent with the previous simulation and experiment. From the experimental results in Figure 18, it can be seen that the trained detector and the proposed controller achieve better prediction and control capabilities.

## 6. Conclusions

In this paper, a lightweight steering-angle prediction network model based on YOLOv5 and an adaptive output feedback control scheme with output constraints based on neural networks has been introduced to regulate the predicted steering angle of YOLOv5Ms effectively. We used YOLOv5Ms as a detector to solve the challenging task of steering-angle prediction in this paper. Meanwhile, an adaptive output feedback control scheme with output constraints based on neural networks has been proposed to ensure that the steering system responds quickly and accurately to the desired steering-angle signal. To train the YOLOv5Ms detector and enhance the generalization capability of the proposed detection model in this study, we conducted an extended data-collection experiment at Western Xia Park in Yinchuan City, building upon our previously created lane-line data set. The images of the data sets were labeled one by one by comparing the steering angle collected in the videos. The accuracy and response speed of the detector can meet the actual requirements after real-time testing. Furthermore, the proposed controller can improve the convergence of the tracking error and eliminate the influence of disturbance. Meanwhile, the proposed controller enhances the steering control accuracy of the steering angle predicted via YOLOv5Ms. The experimental results show that the trained network and the proposed controller perform well. The research in this paper can be further extended to other aspects. Firstly, due to the limited experimental conditions, the prediction and tracking of the angle are only experimented in the SbW experimental bench, and they will be experimentally verified in the actual car in the future. In the controller design, only angle tracking was considered, and the angle tracking of the SbW system will be combined with the trajectory tracking control of the AV in the future.

## Figures and Tables

**Table 1:** Specifications for YOLOv5Ms of the backbone.

Input	Operator	Size	♯ Out	SE	NL	s
$640^{2}\times 3$	2d, 3×3	−	16	−	HS	2
$320^{2}\times 16$	bk, 3×3	16	16	✓	RE	2
$160^{2}\times 16$	bk, 3×3	72	24	−	RE	2
$80^{2}\times 24$	bk, 3×3	88	24	−	RE	1
$80^{2}\times 24$	bk, 5×5	96	40	✓	HS	2
$40^{2}\times 40$	bk, 5×5	240	40	✓	HS	1
$40^{2}\times 40$	bk, 5×5	240	40	✓	HS	1
$40^{2}\times 40$	bk, 5×5	120	48	✓	HS	1
$40^{2}\times 48$	bk, 5×5	144	48	✓	HS	1
$40^{2}\times 48$	bk, 5×5	288	96	✓	HS	2
$20^{2}\times 96$	bk, 5×5	576	96	✓	HS	1
$20^{2}\times 96$	bk, 5×5	576	96	✓	HS	1

**Table 2:** Specifications for YOLOv5Ml of the backbone.

Input	Operator	Size	♯ Out	SE	NL	s
$640^{2}\times 3$	2d, 3×3	−	16	−	HS	2
$320^{2}\times 16$	bk, 3×3	16	16	−	RE	1
$320^{2}\times 16$	bk, 3×3	64	24	−	RE	2
$160^{2}\times 24$	bk, 3×3	72	24	−	RE	1
$160^{2}\times 24$	bk, 5×5	72	40	✓	RE	2
$80^{2}\times 40$	bk, 5×5	120	40	✓	RE	1
$80^{2}\times 40$	bk, 5×5	120	40	✓	RE	1
$80^{2}\times 40$	bk, 3×3	240	80	−	HS	2
$40^{2}\times 80$	bk, 3×3	200	80	−	HS	1
$40^{2}\times 80$	bk, 3×3	184	80	−	HS	1
$40^{2}\times 80$	bk, 3×3	184	80	−	HS	1
$40^{2}\times 80$	bk, 3×3	480	112	✓	HS	1
$40^{2}\times 112$	bk, 3×3	672	112	✓	HS	1
$40^{2}\times 112$	bk, 5×5	672	160	✓	HS	2
$20^{2}\times 112$	bk, 5×5	960	160	✓	HS	1
$20^{2}\times 160$	bk, 5×5	960	160	✓	HS	1

**Table 3:** Lane-turning categories and steering angles.

Classes	L60	L45	L35	L25	L15	L10	L05	G	R05	R10	R15	R25	R35	R45	R60
**Angle**	−60	−45	−35	−25	−15	−10	−5	0	5	10	15	25	35	45	60
**Label**	14	13	12	11	10	9	8	0	1	2	3	4	5	6	7

**Table 4:** Details of training process and results.

Model			Loss ($\times 10^{-3}$)					mAP (%)		
	Params (×10^6^)	Weight (M)	$T_{C}^{1}$	$T_{O}^{1}$	$T_{\mathrm{cl}}^{1}$	Pre (%)^2^	Rec (%)^2^	$m_{0.5}^{2}$	$m_{.5:.95}^{2}$	*T*(*h*)^2^
YOLOv5Ms	5.07	10.1	19.80	11.57	5.71	95.83	96.28	99.10	74.60	16.67
YOLOv5Ml	17.85	36.2	18.82	10.51	3.54	97.32	97.93	99.42	76.89	25.95
YOLOv5s	7.05	14.5	19.59	12.61	7.55	89.24	90.09	98.69	69.57	21.86
YOLOv5m	20.93	40.3	15.52	9.18	3.32	99.05	99.09	99.49	79.16	41.67
YOLOv5l	46.19	88.7	14.49	7.85	2.71	99.22	99.40	99.41	86.59	58.09
YOLOv5x	86.32	173.3	13.38	6.59	2.14	99.50	99.64	99.42	91.85	76.27

^1^ $T_{C}$, $T_{O}$, and $T_{cl}$: represent each error of training, respectively. ^2^ Pre: precision; Rec: recall; $m_{0.5}$: mAP@0.5; $m_{.5:.95}$: mAP@0.5:0.95; T(h): training time (hours).

**Table 5:** Parameters for SbW system.

Parameters	Value
$J_{fw}$ (kg· $m^{2}$)	3.8
$J_{sm}$ (kg· $m^{2}$)	0.0045
$B_{fw}$ (Nms/rad)	10
$B_{sm}$ (Nms/rad)	0.05
$i_{mc}$	18

## Data Availability

Data are contained within the article.

## Abbreviations

AVs	Autonomous vehicles	GIoU	Generalized intersection
SbW	Steer by wire	CNN	Convolutional neural network
WHO	World Health Organization	DL	Deeping learning
MT-LfD	Multi-task demonstration learning	SE	Squeeze and excitation
HS	h-swish	ReLU	Rectified linear unit
FCs	Fully connected layers	MSE	Mean square error
BBOX	Bounding box	IoU	Intersection over union
DIoU	Distance-IoU	CIoU	Comprehensive IoU
EIoU	Efficient version of IoU	AP	Average precision
TP	True positive	FP	False positive
FN	False negative	AMD	Advanced Micro Devices
CAN	Controller area network	RBFNN	Radial basis function neural network
BSC	Backstepping control method	HIL	Hardware-in-the-loop
NAS	Network architecture	RCNN	Region with CNN
SSD	Single Shot MultiBOX Detector	YOLO	You Only Look Once
YOLOv5Ms	You Only Look Once version 5 with MobilNet version 3		

## Appendix A

The algorithm followed in this study is presented in Algorithm A1.

**Algorithm A1** Steering Angle prediction and control
**Require:** Input image sequences **Ensure:** Controlled motor position 1. nput_size = (640, 640, 3), nc= 15, anchors = [(10, 13), (16, 30), (33, 23), (30, 61), (62, 45), (59, 119), (116, 90), (156, 198), (373, 326)], numanchors = len(anchors) 2. Input picture: Image = read_image(path) 3. Image preprocess: Processed_image = preprocess_image(Image) 4. Loading model: Model = load_model(’YOLOv5Ms’) 5. Image reasoning: Outputs = Model(Processed_image) 6. Analytical prediction result: Predictions = Parse_predictions(Outputs) 7. Filter prediction result: Filtered_predictions = Filter_predictions(Predictions) 8. Output the final prediction result to controller: Final prediction result=Ym 9. Estimate angular velocity, model uncertainty, and external disturbance: ${\hat{x}}_{2}$, ${\hat{\tau }}_{ef}$, ${\hat{\tau }}_{D}$. 10. Adaptive neural network output feedback controller provides output $\tau_{m}$, and controls motor position based on this controller output. 11. Adjust motor position. 12. **return** Controlled motor position

## References

[1] Elallid B.B., Benamar N., Hafid A.S., Rachidi T., Mrani N. (2022). A Comprehensive Survey on the Application of Deep and Reinforcement Learning Approaches in Autonomous Driving. J. King Saud Univ.-Comput. Inf. Sci..

[2] Fagnant D.J., Kockelman K. (2015). Preparing a nation for autonomous vehicles: Opportunities, barriers and policy recommendations. Transp. Res. Part A Policy Pract..

[3] Gidado U.M., Chiroma H., Aljojo N., Abubakar S., Popoola S., Mohammed A. (2020). A survey on deep learning for steering angle prediction in autonomous vehicles. IEEE Access.

[4] Peng B., Sun Q., Li S.E., Kum D., Yin Y., Wei J., Gu T. (2021). End-to-end autonomous driving through dueling double deep Q-network. Automot. Innov..

[5] Ma Y., Wang Z., Yan G., Yang L. (2020). Artificial intelligence applications in the development of autonomous vehicles: A survey. IEEE/CAA J. Autom. Sin..

[6] Tampuu A., Matiisen T., Semikin M., Fishman D., Muhammd N. (2022). A Survey of End-to-End Driving: Architectures and Training Methods. IEEE Trans. Neural Netw. Learn. Syst..

[7] Yurtsever E., Lambert J., Carballo A., Takeda K. (2020). A Survey of Autonomous Driving: Common Practices and Emerging Technologies. IEEE Access.

[8] Russakovsky O., Deng J., Su H., Krause J., Satheesh S., Ma S., Huang Z., Karpathy A., Khosla A. (2015). Imagenet large scale visual recognition challenge. Int. J. Comput. Vision.

[9] Polack P., Altch F., Andr B., Fortelle A. The kinematic bicycle model: A consistent model for planning feasible trajectories for autonomous vehicles?. Proceedings of the 2017 IEEE Intelligent Vehicles Symposium (IV).

[10] Mohammed M.S., Abduljabar A.M., Faisal M.M., Mahmmod B.M., Abdulhussain S.H., Khan W., Liatsis P., Hussain A. (2023). Low-cost autonomous car level 2: Design and implementation for conventional vehicles. Results Eng..

[11] Pomerleau D.A. (1988). Alvinn: An autonomous land vehicle in a neural network. Adv. Neural Inf. Process. Syst..

[12] Muller U., Ben J., Cosatto E., Flepp B., Cun Y. Off-road obstacle avoidance through end-to-end learning. Proceedings of the Advances in Neural Information Processing Systems.

[13] Bojarski M., Del T.D., Dworakowski D., Firner B., Flepp B., Goyal P., Jackel L.D. (2016). End to end learning for self-driving cars. arXiv.

[14] Mohseni F., Voronov S., Frisk E. (2018). Deep learning model predictive control for autonomous driving in unknown environments. IFAC-PapersOnLine.

[15] Mehta A., Subramanian A. Learning end-to-end autonomous driving using guided auxiliary supervision. Proceedings of the 11th Indian Conference on Computer Vision, Graphics and Image Processing.

[16] Zeng W., Luo W., Suo S., Sadat A., Yang B., Casas S., Urtasun R. End-to-end interpretable neural motion planner. Proceedings of the IEEE/CVF Conference on Computer Vision and Pattern Recognition.

[17] Le M., Yi D., Dianati M., Mouzakitis A. (2022). A survey on imitation learning techniques for end-to-end autonomous vehicles. IEEE Trans. Intell. Transp. Syst..

[18] He K., Zhang X., Ren S., Sun J. Deep residual learning for image recognition. Proceedings of the IEEE Conference on Computer Vision and Pattern Recognition.

[19] Karadeniz A.M., Ballagi Á., Kóczy L.T. (2024). Transfer Learning-Based Steering Angle Prediction and Control with Fuzzy Signatures-Enhanced Fuzzy Systems for Autonomous Vehicles. Symmetry.

[20] Ren S., He K., Girshick R., Sun J. (2015). Faster r-cnn: Towards real-time object detection with region proposal networks. Adv. Neural Inf. Process. Syst..

[21] Bochkovskiy A., Wang C., Liao H.M. (2020). Yolov4: Optimal speed and accuracy of object detection. arXiv.

[22] Ye C., Wang Y., Wang Y., Tie M. (2022). Steering angle prediction yolov5-based end-to-end adaptive neural network control for autonomous vehicles. Proc. Inst. Mech. Eng. Part D J. Automob. Eng..

[23] Dong X., Yan S., Duan C. (2022). A lightweight vehicles detection network model based on yolov5. Eng. Appl. Artif. Intell..

[24] Marumo Y., Nagai M. (2007). Steering control of motorcycles using steerby-wire system. Veh. Syst. Dyn..

[25] Setlur P., Wagner J.R., Dawson D.M., Braganza D. (2006). A trajectory tracking steer-by-wire control system for ground vehicles. IEEE Trans. Veh. Technol..

[26] Huang C., Naghdy F., Du H. (2018). Delta operatorbased fault estimation and fault-tolerant model predictive control for steer-by-wire systems. IEEE Trans. Control Syst. Technol..

[27] Sun Z., Zheng J., Man Z., Wang H. (2016). Robust control of a vehicle steer-by-wire system using adaptive sliding mode. IEEE Trans. Control Syst. Technol..

[28] Ye M., Wang H. (2020). Robust adaptive integral terminal sliding mode control for steer-by-wire systems based on extreme learning machine. Comput. Electr. Eng..

[29] Chen J., Guo Y. (2024). Design and Non-Linearity Optimization of a Vertical Brushless Electric Power Steering Angle Sensor. Sensors.

[30] Garcia G., Molina C., Luque B., Rafael M., Juan M. (2021). Road pollution estimation from vehicle tracking in surveillance videos by deep convolutional neural networks. Appl. Soft Comput..

[31] Yao J., Qi J., Zhang J., Shao H., Yang J., Li X. (2021). A real-time detection algorithm for kiwifruit defects based on yolov5. Electronics.

[32] Howard A.G., Zhu M., Chen B., Kalenichenko D., Wang W., Wey T., Andreetto M., Adam H. (2017). Mobilenets: Efficient convolutional neural networks for mobile vision applications. arXiv.

[33] Sandler M., Howard A., Zhu M., Zhmoginov A., Chen L.C. Mobilenetv2: Inverted residuals and linear bottlenecks. Proceedings of the IEEE Conference on Computer Vision and Pattern Recognition.

[34] Zhao L., Wang L. (2022). A new lightweight network based on mobilenetv3. KSII Trans. Internet Inf. Syst..

[35] Gu J., Wang Z., Kuen J., Ma L., Shahroudy A., Shuai B., Liu T., Wang X., Wang G., Cai J. (2018). Recent advances in convolutional neural networks. Pattern Recognit..

[36] Krizhevsky A., Sutskever I., Hinton G.E. (2012). Imagenet classification with deep convolutional neural networks. Adv. Neural Inf. Process. Syst..

[37] Niu Z., Zhong G., Yu H. (2021). A review on the attention mechanism of deep learning. Neurocomputing.

[38] Guo M.H., Xu T.X., Liu J.J., Liu Z.N., Jiang P.T., Mu T.J., Zhang S.H., Martin R.R., Cheng M.M., Hu S.M. (2022). Attention mechanisms in computer vision: A survey. Comput. Vis. Media.

[39] Hu J., Shen L., Sun G. Squeeze-and-excitation networks. Proceedings of the IEEE Conference on Computer Vision and Pattern Recognition.

[40] Redmon J., Farhadi A. (2018). Yolov3: An incremental improvement. arXiv.

[41] Yu J., Jiang Y., Wang Z., Cao Z., Huang T. Unitbox: An advanced object detection network. Proceedings of the 24th ACM international conference on Multimedia.

[42] Rezatofighi H., Tsoi N., Gwak J., Sadeghian A., Reid I., Savarese S. Generalized intersection over union: A metric and a loss for bounding box regression. Proceedings of the IEEE/CVF Conference on Computer Vision and Pattern Recognition.

[43] Zheng Z., Wang P., Liu W., Li J., Ye R., Ren D. (2020). Distance-iou loss: Faster and better learning for bounding box regression. Proc. AAAI Conf. Artif. Intell..

[44] Zhang Y.F., Ren W., Zhang Z., Jia Z., Wang L., Tan T. (2022). Focal and efficient iou loss for accurate bounding box regression. Neurocomputing.

[45] Gevorgyan Z. (2022). Siou loss: More powerful learning for bounding box regression. arXiv.

[46] Zhang H., Zhang S. (2023). Shape-iou: More accurate metric considering bounding box shape and scale. arXiv.

[47] Visa S., Ramsay B., Ralescu A.L., Van Der Knaap E. (2011). Confusion matrix-based feature selection. Maics.

[48] Na J., Chen Q., Ren X., Guo Y. (2014). Adaptive prescribed performance motion control of servo mechanisms with friction compensation. IEEE Trans. Ind. Electron..

[49] Kim S. (2019). Moment of inertia and friction torque coefficient identification in a servo drive system. IEEE Trans. Ind. Electron..

[50] Liu K., Zhu Z. (2017). Determination of moment of inertia of permanent magnet synchronous machine drives for design of speed loop regulator. IEEE Trans. Ind. Electron..

[51] Deng H., Krstic M. (1997). Stochastic nonlinear stabilization-i: A backstepping design. Syst. Control Lett..

[52] Li Y., Qiang S., Zhuang X., Kaynak O. (2004). Robust and adaptive backstepping control for nonlinear systems using rbf neural networks. IEEE Trans. Neural Netw..

[53] Chen M., Ge S. (2013). Direct adaptive neural control for a class of uncertain non-affine nonlinear systems based on disturbance observer. IEEE Trans. Cybern..

[54] Peng Z., Wang J., Wang D. (2017). Distributed containment maneuvering of multiple marine vessels via neurodynamics-based output feedback. IEEE Trans. Ind. Electron..

